# *C*_2_‐Symmetric Pseudopeptidic Schiff Bases Drive Discrete M_2_L_2_ Complexes: Coordination, Stability, and Photophysical Response

**DOI:** 10.1155/bca/1063983

**Published:** 2026-09-23

**Authors:** Mrituanjay D. Pandey, Arpna Tamrakar, Vicente Martí-Centelles, Armando Ferrer, Eduardo García-Verdugo, Santiago V. Luis, Belén Altava

**Affiliations:** ^1^ Department of Chemistry, Institute of Science, Banaras Hindu University, Varanasi, India, bhu.ac.in; ^2^ Department of Inorganic and Organic Chemistry, University Jaume I, Castellón, Spain; ^3^ Instituto Interuniversitario de Investigación de Reconocimiento Molecular y Desarrollo Tecnológico (IDM), Universitat Politècnica de València, Universitat de València, Valencia, Spain, upv.es; ^4^ Departamento de Química, Universitat Politècnica de València, Valencia, Spain, upv.es; ^5^ Department of Organic Chemistry, Institute of Chemistry, Federal University of Bahia, Salvador, Bahía, Brazil, ufba.br; ^6^ Department of Chemistry, Faculty of Natural and Exact Sciences, University of Oriente, Santiago de Cuba, Cuba, uo.edu.cu

**Keywords:** C_2_-symmetric pseudopeptides, dinuclear M_2_L_2_ complexes, salicylaldimine (Schiff base) ligands, zinc(II) and copper(II) coordination

## Abstract

We report the design and synthesis of two new *C*_2_‐symmetric, multifunctional pseudopeptidic ligands derived from *L*‐valine and *L*‐phenylalanine that integrate dual Schiff‐base units (one per amino‐acid fragment) and an aromatic central spacer. These ligands were rationally engineered to favor the self‐assembly of discrete M_2_L_2_ architectures by combining *C*_2_ symmetry with preorganized, convergent donor sets. Their metal‐binding behavior, in particular toward Zn(II) and Cu(II), was elucidated by potentiometric titrations, UV–Vis spectrophotometry, and single‐crystal X‐ray diffraction. The combined data demonstrate a strong propensity to form dimeric M_2_L_2_ species, with particularly pronounced stabilization for Zn(II). X‐ray analysis of the Zn_2_L_2_ complex reveals a pseudo‐octahedral environment in which each Zn(II) is coordinated by imine nitrogen donors together with amide and phenolate oxygen atoms, with the required donor set completed cooperatively by two different ligand subunits, thus enforcing the targeted dimeric topology. Complex formation is accompanied by fluorescence enhancement for Zn(II), marked changes in the circular dichroism signatures of the Schiff‐base chromophores, and characteristic d–d transition bands for Cu(II) and Ni(II) complexes. These results establish a modular pseudopeptidic platform for programming discrete M_2_L_2_ metallosupramolecular assemblies and their associated chiroptical and emissive responses.

## 1. Introduction

Schiff bases and their derivatives are highly versatile ligands [1–4]. Their metal complexes are of great interest due to their reported applications such as those in catalysts [5–7], new materials [8–10] receptors for anions [11], or in biochemistry [12–14]. Much research has been focused on the design of multidentate Schiff‐base ligands able to form binuclear complexes by the introduction of additional donor atoms, allowing the modification of the metal ion coordination environment [15–18]. In this regard, binuclear complexes have attracted immense attention due to their biological relevance, as multiple binding/coordination sites are present in many enzymes [19–21]. Furthermore, from a biological point of view, zinc and copper are important trace elements playing vital roles in various biological and physiological processes. Thus, for example, copper is responsible for the normal function of many tissues, including the immune, nervous, and cardiovascular systems [22–25], and zinc plays vital roles in enzyme catalysis, apoptosis, and neurotransmission [26–28]. Moreover, Zn(Salen)‐type complexes have emerged as relevant targets, for example for their fluorescence properties as chemosensors [29–31].

**FIGURE 1:**
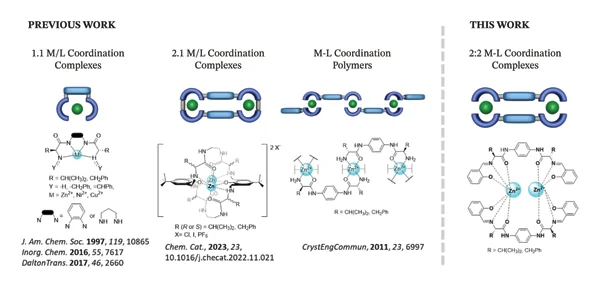
Different metal coordination models for the pseudopeptidic ligands reported by our group.

In this context, the use of amino acids as building blocks for the design of Schiff base structures can be considered an important strategy, not only for the ability of amino acids to bind metal ions but also because they provide coordination environments like those found in metalloproteins. Thus, for instance, Polt and coworkers, in their seminal work in this area, explored the catalytic activity of a series of symmetrical tetracoordinated Schiff base metal complexes derived from pseudopeptides [32–34]. In this field, our group has studied the coordination ability of several *C*_2_‐symmetrical open‐chain bis(amino amides) derived from amino acids toward different ions, their behavior being modulated by the spacer linking the two amino acid moieties, the nature of the amino‐acid component, and the presence of substituents at the terminal amino group [35–39]. In these ligands, each coordinating subunit can contribute with different donor atoms: the amine nitrogen atom, the amide nitrogen atom when this group is deprotonated and the oxygen of the carbonyl group. However, only two of those donors can coordinate simultaneously to the same metal ion. Although a variety of coordination arrangements are possible (Figure 1), it has been found that if the spacer allows it, the convergence of the two subunits onto a single metal ion in a square planar arrangement is favored. Thus, the most straightforward approach to achieve the assembly of two ligands and two metal ions into discrete M_2_L_2_ complexes is the modification of the spacer, making it very short or very long and rigid, to make the convergence of the two pseudopeptidic subunits impossible. The tendency of Cu^2+^ ions to form square planar complexes suggested that this target could be easier with this ion. However, the formation of an M_2_L_2_ structure has been rather elusive up to now, despite its potential applications in different fields [40], and was only observed for the shortest (ethylenic) spacer although the actual structure in the crystal was that of a coordination polymer in which the M_2_L_2_ units are connected through O–Cu bonds [35]. This target was even more difficult for Zn^2+^ ions because of their higher coordination numbers, as evidenced by the formation of coordination polymers with Zn^2+^:ligand ratios of 1:1, 1:1.33, and 1:1.5 when using pseudopeptides based on a rigid paraphenylene spacer [37]. Only in the case of macrocyclic pseudopeptidic polyfunctional ligands, discrete dinuclear Zn^2+^ complexes have been obtained [40]. In light of those results, a new approach was considered with the design of *C*_2_‐symmetrical open‐chain pseudopeptides combining a rigid *p*‐phenylene spacer with the presence of additional donor atoms for the assembly of discrete M_2_L_2_ complexes.

Here, we present the synthesis and characterization of a new family of *C*_2_‐pseudopeptidic Salen‐type Schiff‐base ligands derived from *L*‐valine and *L*‐phenylalanine and featuring a *p*‐diaminobenzene aromatic spacer, and we establish their metal‐binding behavior primarily through a comprehensive potentiometric and spectroscopic study [41, 42]. In particular, UV–Vis absorption, fluorescence emission (including turn‐on response), and circular dichroism (CD) provide a detailed optical readout of complex formation with Cu(II) and Zn(II). This metallomacrocyclic coordination mode is new for pseudopeptidic ligands, underscoring how ligand geometry and donor‐atom disposition govern the final structural outcome. The observed assembly of the M_2_L_2_ macrocycles reveals how an appropriate design for a favorable preorganization of the ligands can bias all the possible combinations of the M_n_L_m_ coordination complexes toward the formation of the M_2_L_2_ species under equilibrium conditions. Moreover, Zn(II) complexes display fluorescence turn‐on behavior, highlighting the potential of these scaffolds as optical probes for Zn(II).

## 2. Experimental Section

### 2.1. General Experimental Details

Spectral measurements were carried out at 25°C. UV–Vis spectra were recorded on a Hewlett–Packard 8453 spectrophotometer and fluorescence spectra were recorded on a RF‐5301 Spectrophotometer with a 10‐mm quartz cell or on a Spex Fluorolog [3–11] instrument equipped with a 450‐W xenon lamp (right‐angle mode). CD spectra were recorded with a JASCO J‐810 spectropolarimeter. FT‐IR spectra were acquired on a JASCO 6200 equipment with a MIRacle single reflection ATR diamond/ZnSe accessory. Melting points were measured using a standard melting point apparatus and are uncorrected. ^1^H and ^13^C‐NMR spectra were obtained on a Varian INOVA 500 spectrometer (500 MHz for ^1^H and 125 MHz for ^13^C) or on a Bruker Advance III HD 300 spectrometer (300 MHz for ^1^H and 75 MHz for ^13^C). Chemical shifts are reported in ppm using TMS as the reference. Electrospray ionization high resolution mass spectra (ESI‐HRMS) were recorded on a MICRO MASS QUATTRO II triple quadruple mass spectrometer. Elemental analyses (C, N, and H) were recorded on an EXTER CE‐440 CHN analyzer. Compounds **1** were synthesized as previously reported [35].

Single crystals of **3a** (C_60_H_64_N_4_O_8_Zn_2_) were grown from acetonitrile via slow evaporation. A suitable crystal was selected and mounted on an X‐ray diffractometer. The crystal was kept at 293(2) K during data collection. Using Olex2 [43], the structure was solved with the SHELXS structure solution program using Direct Methods and refined with the SHELXL refinement package using least squares minimization [44].

Crystal data for **3a** C_60_H_64_N_8_O_8_Zn_2_ (*M* = 1155.98 g/mol): trigonal, space group P3_1_21 (no. 152), a = 18.9710(7) Å, c = 28.1733(11) Å, V = 8781.1(6) Å3, Z = 3, T = 293(2) K, *μ*(MoKα) = 0.446 mm‐1, Dcalc = 0.728 g/cm^3^, 63027 reflections measured (6.1 ≤ 2Θ ≤ 58.8), 14687 unique (Rint = 0.1156, Rsigma = 0.1538), which were used in all calculations. The final R1 was 0.0923 (> 2sigma(I)) and wR2 was 0.2629 (all data). The crystal exhibited limited diffraction quality, which is reflected in a relatively low ratio of observed to unique reflections and elevated agreement factors (Rint, R1, and wR2). Additionally, the crystal lattice contains very large solvent‐accessible voids occupied by highly disordered solvent molecules. This structural feature results in an unusually low calculated density. Because the residual electron density corresponding to this disordered solvent could not be reasonably modeled with discrete atomic positions, the solvent‐masking algorithm SQUEEZE (PLATON) was employed [45]. Consequently, the reported moiety formula reflects the main complex alongside masked solvent. It is important to emphasize that while the poor crystal quality inherently limits the metric precision of individual bond lengths and angles, the overall structural connectivity, the binuclear nature of the complex, and the coordination geometry around the metal centers remain completely unambiguous.

### 2.2. Electromotive Force Measurements

Potentiometric titrations were carried out at 298.1 ± 0.1 K using 0.1‐M NaCl as the supporting electrolyte to maintain a constant ionic strength and a standard experimental setup [46]. The emf data were obtained with a Crison equipment using the provided computer program CrisonCapture for data acquisition. A reference Ag/AgCl electrode in saturated KCl solution was used, and the glass electrode was calibrated as a hydrogen‐ion concentration probe by titrating previously standardized amounts of HCl with CO_2_‐free NaOH solutions. The equivalence point was determined through the Gran’s method [47, 48], providing the standard potential (E°′) and the ionic product of pure water (13.78). For the selected H_2_O/CH_3_CN mixture used (7/3, v/v), this value was calculated as 14.6, in agreement with reported values [49]. The software HYPERQUAD was used for calculating protonation and stability constants [50], and the HySS [29] was used to obtain the distribution diagrams [51]. The pH range investigated was 3.5–11.0 to maintain the stability of the solution, and the concentration of both metal ions and the ligands 0.1 mM, with 1:1 M^2+^:L molar ratios. At least two titration curves were obtained for each system and treated as a single set or as separated curves without significant variations in the values for the association constants. These sets of data were merged and treated simultaneously to give the final stability constants. For each titration curve, between 125 and 201 experimental points were recorded and subsequently used in the fitting procedure.

### 2.3. Synthesis of Compound 2a

Compound **1a** (0.612 g, 2 mmol) was dissolved in methanol (10 mL). Once a clear solution was obtained, salicylaldehyde (0.42 mL, 4 mmol) previously dissolved in methanol, was added. The reaction mixture was stirred under reflux for 5 h. The solvent was then vacuum evaporated, and the residue washed with diethyl ether. The crude product was recrystallized via slow evaporation from CH_2_Cl_2_/acetonitrile. Yield (0.78 g, 68%); mp 229–230^o^C; [*α*]^25^_D_ = +191.90 (*c* 0.01 g/5 mL, CH_2_Cl_2_); FT‐IR: 751, 840, 1228, 1311, 1540, 1659, 2965 cm‐1; ^1^H NMR (400 MHz, CD_3_OD) (*δ*): 12.44 (s, 2H), 8.37 (s, 2H), 7.72 (s, 2H), 7.50 (s, 4H), 7.46 – 7.30 (m, 4H), 7.09 – 6.92 (m, 4H), 3.80 (d, J = 4.1 Hz, 2H), 2.66 – 2.44 (m, 2H), 1.00 (dd, J = 19.0, 6.9 Hz, 12H) ppm. ^13^C NMR (101 MHz, CD_3_OD) (*δ*): 169.25, 168.42, 160.79, 133.90, 133.68, 132.46, 120.88, 119.64, 118.56, 117.34, 80.21, 32.64, 19.81, 17.49 ppm. HRMS (Q‐TOF, MS ESI+) m/z: Calculated for C_30_H_35_N_4_O_4_^+^ [2a + H]^+^: 515.27; Found: 515.27 (87%). Calculated for C_30_H_36_N_4_O_4_^2+^ [**2a**+2H]^2+^: 258.14; Found: 258.1353 (100%). HRMS (Q‐TOF, MS ESI^+^) m/z: Calculated for C_30_H_33_N_4_O_4_^−^ [**2a**‐H]^−^: 513.25; Found: 513.2510 (100%).

### 2.4. Synthesis of Compound 2b

Compound **1b** (0.402 g, 1 mmol) was dissolved in methanol (10 mL). Once a clear solution was obtained, salicylaldehyde (0.10 mL, 2 mmol) previously dissolved in methanol, was added. The reaction mixture was stirred under reflux for 5 h. The solvent was then vacuum evaporated, and the residue washed with diethyl ether. Yield (0.43 g, 70%); Mp. 218°C–220°C, [*α*]^25^_D_ = −105.80 (*c* 0.002 g/mL, CH_2_Cl_2_). FT‐IR: 697, 757, 818, 1084, 1274, 1311, 1407, 1490, 1518, 1626, 1670, 3023, 3364 cm‐1. ^1^H NMR (500 MHz, CDCl_3_) (*δ* ppm): 3.11 (2 H, dd, J = 8.9, 13.6 Hz), 3.42 (2 H, dd, J = 3.6, 13.6 Hz), 4.08 (2 H, dd, J = 3.6, 8.9 Hz), 6.82 (2 H, td, J = 1.0, 7.5 Hz), 6.92 – 6.97 (2 H, m), 7.04 – 7.20 (12 H, m), 7.30 (2 H, ddd, J = 1.7, 7.3, 8.4 Hz), 7.39 (4 H, s), 7.63 (2 H, s), 7.88 (2 H, s), 12.10 (2 H, s, Ar‐OH) ppm. ^13^C NMR (75 MHz, CDCl_3_) (*δ* ppm): 41.1, 75.9, 117.1, 118.2, 119.4, 120.8, 126.9, 128.5, 129.7, 132.2, 133.5, 133.7, 136.6, 160.4, 168.1, 168.7 ppm. HRMS (Q‐TOF, MS ESI^+^) m/z: Calculated for C_38_H_35_N_4_O_4_^+^ [**2b** + H]^+^: 611.27; Found: 611.2654 (100%); Calculated for C_38_H_36_N_4_O_4_^2+^ [**2b**+2H]^2+^: 306.13; Found: 306.1339 (40%).

### 2.5. Synthesis of the Zn_2_L_2_ Complex for Ligand 2a (3a)

Compound **2a** (0.257 g, 0.5 mmol) was dissolved in methanol/chloroform (1:1) (20 mL) and then triethylamine (0.5 mL) was added. To the clear solution formed, the stoichiometric amount of zinc chloride (0.068 g, 0.5 mmol) was further added. The mixture was stirred for 3 h at constant stirring. The precipitate formed was filtered and washed with diethyl ether. Yield (0.75 g, 65%); Mp. 229°C–230°C; FT‐IR: 736, 757, 836, 901, 1026, 1149, 1192, 1228, 1447, 1465, 1515, 1536, 1622, 1738, 2926, 2969, 3274 cm^−1^. ^1^H NMR (400 MHz, CD_3_OD) (*δ*): 8.30 (s, 2H), 7.28 (dd, J = 7.9, 1.8 Hz, 2H), 7.22 (ddd, J = 11.3, 6.6, 3.1 Hz, 2H), 7.10 (s, 4H), 6.72 (d, J = 7.9 Hz, 2H), 6.63 – 6.54 (m, 2H), 3.54 (d, J = 9.2 Hz, 2H), 2.41 – 2.28 (m, 2H), 1.02 (d, J = 6.6 Hz, 6H), 0.87 (d, J = 6.8 Hz, 6H) ppm. ^13^C NMR (101 MHz, CD_3_OD) (*δ*): 172.05, 171.61, 171.42, 137.34, 135.51, 123.12, 120.26, 119.76, 115.19, 82.67, 49.64, 49.50, 49.43, 49.28, 49.21, 49.00, 48.79, 48.57, 48.36, 33.48, 19.68, 19.56 ppm. HRMS (Q‐TOF, MS ESI^+^) m/z: Calculated for C_60_H_65_N_8_O_8_Zn_2_^+^ [**3a** + H]^+^: 1157.35; Found: 1157.3495 (50%). Calculated for C_60_H_66_N_8_O_8_Zn_2_^2+^ [**3a**+2H]^2+^: 579.17; Found: 579.1754 (100%).

## 3. Results and Discussion

### 3.1. Synthesis, Characterization, and Acid–Base Properties of the Ligands

**SCHEME 1:**
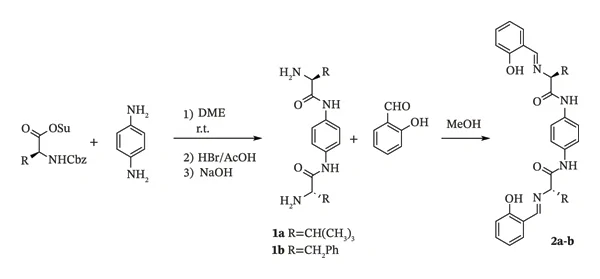
General synthetic protocol for ligands **2a-b**.

Salen‐type pseudopeptides **2a-b** derived from L‐phenylalanine and L‐valine were obtained from the corresponding bis(amino amides) **1a-b** by reaction in MeOH with o‐hydroxybenzaldehyde. Bis(amino amides) **1a-b** were prepared starting from the corresponding N‐Cbz‐protected amino acid N‐hydroxysuccinimide esters, via coupling with 1,4‐diaminobenzene and the subsequent N‐deprotection using HBr/AcOH, following previously reported procedures (Scheme 1) [37]. The final ligands **2a**‐**b** were fully characterized using standard techniques (^1^H NMR, ^13^C NMR, FT‐IR, and MS, see ESI Figures S9–S14).

A prerequisite to understanding the coordination behavior of any ligand is to have an insight into the acid–base properties of the ligand under consideration [52–54]. Potentiometric studies of Schiff bases related to **2** are often limited by different factors [55]. A first element is their low solubility in water, which is usually solved with the use of mixed organic:water solvents [56]. Thus, potentiometric titrations were performed for ligands **2** in a 0.1‐M aqueous NaCl/CH_3_CN solution (7:3 v/v) at 25°C. On the other hand, imine groups can undergo hydrolysis in aqueous media and, in the case of ditopic compounds like **2**, hydrolysis has been reported under both basic [57, 58] and acidic conditions [59, 60]. Preliminary experiments with the Schiff bases in this study indicated that hydrolysis may occur under strongly acidic conditions (see ESI, Figure S1). Specifically, the ^1^H NMR spectrum of compound **2a** at pH 2.6 (3 mM, HCl in D_2_O/CD_3_CN, 7:3) showed complete imine hydrolysis, evidenced by the disappearance of the signal at 8.4 ppm and the appearance of a new peak at 10 ppm, corresponding to the resulting aldehyde. In contrast, the ^1^H NMR spectrum of the precipitate obtained after treating **2a** in an acidic medium (4 mM, HCl (aq.)/CH_3_CN, 7:3, pH 3) showed minor imine hydrolysis. Based on these findings, pH titrations were performed in the range of 3.5–11.0.

Finally, it must be noted that the presence of tautomeric equilibria (enolimine–ketoamine) adds complexity to the study of these systems. However, such equilibria can only be studied by spectroscopic methods [56, 61, 62]. As they do not affect the protonation state of the ligand, they cannot be analyzed by potentiometry which provides overall protonation constants. In the case of **2a**, initial computational calculations for the neutral H_2_L species suggested that ketoamine conformers were more stable than the enolimine ones (ESI, Figure S2).

**FIGURE 2:**
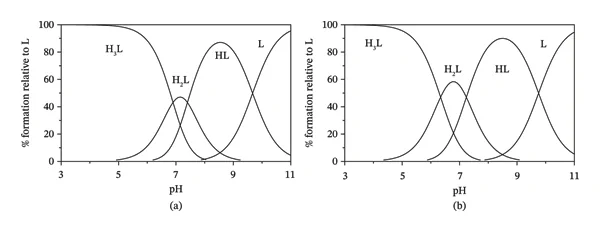
Species distribution diagram **2a** (left) and **2b** (right). Charges are omitted for clarity. NaCl 0.1 M/CH_3_CN (7:3) at 298.1 ± 0.1 K.

The protonation constants and the distribution diagrams obtained for **2a-b** from the potentiometric titration experiments are reported in Table 1 and Figure 2, respectively. For simplicity, **L** has been defined as the bisanionic species obtained after the deprotonation of both phenolic groups in **2**. Although **L** displays four protonation sites, only three protonation constants were detected for both ligands, a behavior frequently reported for ditopic Schiff bases [55]. The assignment of individual sites is not straightforward because these systems can exhibit low‐barrier O···H···N interactions (either in the imine–hydroxy, enamine–oxo, or zwitterionic forms) [63–65], so the first protonation likely involves coupled O/N basicity rather than a single localized site [66–69].

**TABLE 1:** Logarithms of the stepwise protonation constants for **2a** and **2b**.^a^

Equilibria^b^	2a	2b
L + H ⇌HL	9.47 ± 0.01	9.67 ± 0.01
HL + H ⇌ H_2_L	7.66 ± 0.01	7.30 ± 0.01
H_2_L + H ⇌ H_3_L	6.91 ± 0.01	6.38 ± 0.02
Chi^2^	12.97	12.48
Sigma	0.4944	1.7423

^a^NaCl 0.1 M/CH_3_CN (7:3) at 298.1 ± 0.1 K.

^b^Charges omitted for clarity; L refers to the bisdeprotonated ligand.

For **2a** and **2b**, the two most basic steps show log *K* = 9.47/7.66 and 9.67/7.30, respectively, notably lower than values reported for simpler salicylaldehyde/diamine analogs [59, 60, 70–72]. This reduced basicity is consistent with intramolecular hydrogen bonding involving the amide N–H groups and with electrostatic/interaction effects after the first protonation [58]. A third protonation step occurs around neutral pH (log *K* = 6.91 for **2a** and 6.38 for **2b**) associated with the imine fragment. For imine protonation, lower log K values are usually reported for Schiff bases derived from anilines [66–68, 71–73], while higher values are found for aliphatic amines and amino‐acid‐derived Schiff bases [56, 57, 74, 75]. The absence of an experimentally detected fourth protonation constant in these systems has also been noted for other ditopic Schiff bases [71–73], which has been related to strong hydrogen‐bond networks and to monohydrolysis processes activated upon extensive protonation [59, 60]. Overall, the amino‐acid residue has only a minor influence, except for this third step, which is less basic in the phenylalanine derivative **2b**.

The species‐distribution diagrams show that bisdeprotonated species predominate above pH ∼10, whereas H_3_L becomes predominant around pH ∼6 for **2a** and around pH ∼7 for **2b** (Figure 2).

### 3.2. Potentiometric Studies for Zn^2+^ and Cu^2+^ Complexes

Complex formation of ligands **2** with Zn^2+^ and Cu^2+^ ions was studied through potentiometric titrations for a 1:1‐L/M molar ratio in NaCl 0.1 M/CH_3_CN (7:3) at 298.1 ± 0.1 K. Hyperquad refinement of the titration data provided the cumulative stability constants listed in Table 2 [50]. In agreement with the ligand design, dimeric complexes involving two ligands are predominant, consistent with two relatively isolated coordination areas imposed by the rigid spacer. The coordination numbers of Zn^2+^ (∼6) and Cu^2+^ (4‐5, influenced by the Jahn–Teller effect) [76, 77] can facilitate the supramolecular binding of a second ligand to complete the metal coordination.

**TABLE 2:** Logarithms of cumulative constants of complex equilibria for **2a** and **2b** with Zn^2+^ and Cu^2+.^

Species^b^	Equilibria^b^	2a (Val)		2b (Phe)	
		Zn^2+^	Cu^2+^	Zn^2+^	Cu^2+^
MH_6_L_2_	2L + M + 6H ⇌ MH_6_L_2_	58.22 ± 0.12	—	53.85 ± 0.10	—
MH_5_L_2_	2L + M + 5H ⇌ MH_5_L_2_	—	—	47.84 ± 0.06	—
MH_4_L_2_	2L + M + 4H ⇌ MH_4_L_2_	44.59 ± 0.89	—	41.27 ± 0.10	—
MH_3_L_2_	2L + M + 2H ⇌ MH_3_L_2_	37.61 ± 0.92	—	34.33 ± 0.08	—
M_2_H_2_L_2_	2L + 2M + 2H ⇌ M_2_H_2_L_2_	27.93 ± 0.77	38.32 + 0.03	30.89 ± 0.09	37.82 ± 0.03
M_2_HL_2_	2L + 2M + H ⇌ M_2_HL_2_	—	31.08 ± 0.10	—	29.95 ± 0.09
M_2_L_2_	2L + 2M ⇌ M_2_L_2_	21.20 ± 0.27	22.90 ± 0.08	15.07 ± 0.16	21.43 ± 0.07
M_2_L_2_(OH)	2L + 2M + OH ⇌ M_2_L_2_(OH)	13.18 ± 0.13	13.41 ± 0.11	7.37 ± 0.06	11.76 ± 0.09
M_2_L_2_(OH)_2_	2L + 2M + 2OH ⇌ M_2_L_2_(OH)_2_	6.77 ± 0.12	3.67 ± 0.09	−1.37 ± 0.06	1.40 ± 0.09
M_2_L_2_(OH)_4_	2L + 2M + 4OH ⇌ M_2_L_2_(OH)_4_	−10.24 ± 0.11	—		—
Chi^2^		17.52	17.89	13.91	33.47
Sigma		1.025	0.6216	0.5197	0.699

^a^NaCl 0.1 M/CH_3_CN (7:3) at 298 ± 0.1 K.

^b^Charges omitted for clarity.

A marked metal‐dependent speciation is observed. For Cu^2+^, only M_2_L_2_ species with different protonation degrees are detected: Cu_2_L_2_ predominates at basic pH, Cu_2_HL_2_ becomes major around pH ∼8, and Cu_2_H_2_L_2_ dominates under acidic conditions; hydroxylated dinuclear species appear at higher pH. For Zn^2+^, the neutral Zn_2_L_2_ species is only a minor component around pH ∼7 (**2a**) or ∼8 (**2b**). At neutral and acidic pH, mononuclear ZnH_x_L_2_ complexes (still involving two ligands) are predominant and can reach high degrees of protonation, while in basic media, Zn_2_L_2_(OH)_x_ species dominate. This is consistent with the higher flexibility of Zn^2+^ coordination and with the possibility of stabilizing partially protonated assemblies through supramolecular hydrogen bonding between ligands.

Regarding stability (Table 2), cumulative constants for comparable species are generally higher for Cu^2+^ than for Zn^2+^, as commonly observed in coordination chemistry [38]. The amino‐acid residue also modulates stability: for most dimeric M_2_H_x_L_2_ species, constants are higher for the valine derivative **2a** than for the phenylalanine derivative **2b**. This effect is much stronger for Zn^2+^: for the neutral M_2_L_2_ species, the constant is more than five orders of magnitude higher for **2a**, whereas the difference is about one order of magnitude for Cu^2+^. Notably, this trend reverses that reported for related ligands derived from 1,2‐diaminobenzene [37].

**SCHEME 2:**
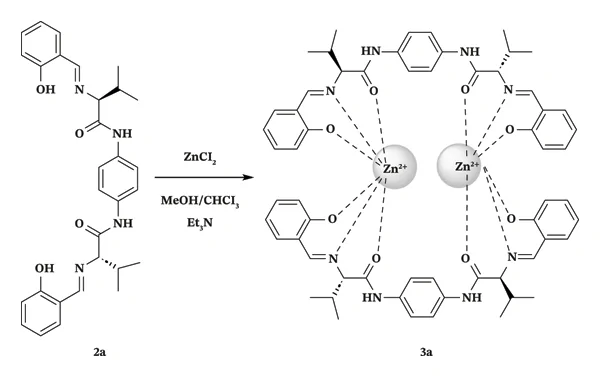
General synthetic protocol for the synthesis of Zn^2+^ complex **3a**.

**FIGURE 3:**
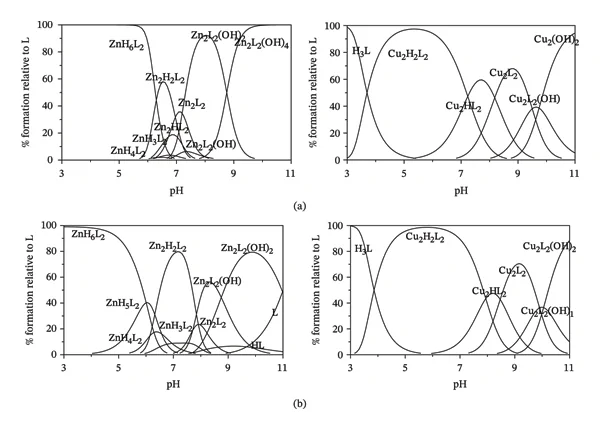
Species distribution diagram of ligands (a) **2a** with Zn^2+^ and Cu^2+^ as a function of pH, 1:1 ligand–metal relationship. (b) **2b** with Zn^2+^ and Cu^2+^ as a function of pH, 1:1 ligand‐to‐metal ratio (0.1 mM each). NaCl 0.1 M/CH_3_CN (7:3) at 298.1 ± 0.1 K (charges omitted of clarity).

In the pseudomacrocyclic M_2_L_2_ organization (Scheme 2), the larger aromatic side chain in **2b** may provide greater shielding of the second coordination sphere from water, decreasing overall stability; this is expected to be more relevant for Zn^2+^. In line with this, hydroxylated Zn_2_L_2_(OH)_x_ species with larger x values are more apparent for **2a** and arise at slightly lower pH than for **2b** (Figure 3). The magnitudes of the stability constants are also consistent with the few examples reported for related dinuclear Schiff base systems [71].

Overall, potentiometry confirms that ligands **2a**‐**b** predominantly form discrete dimeric M_2_L_2_ species with Zn^2+^ and Cu^2+^, with a clear metal‐ and ligand‐dependent speciation and stability pattern. These findings guided the subsequent UV–Vis, fluorescence, and CD studies aimed at further characterizing the complexation behavior.

### 3.3. UV–Vis, Fluorescence, and CD Studies for M^2+^ Complexes

**FIGURE 4:**
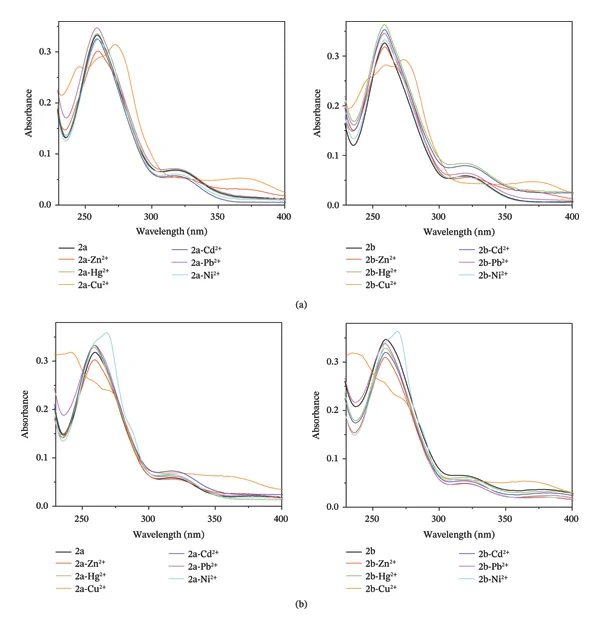
(a) UV–Visible spectra of **2a** and **2b** (10 µM, CH_3_CN/H_2_O 8/2, v/v) in the absence and presence (1:1 molar ratio pH ∼ 6.4) of different metal perchlorates. (b) UV–Visible spectra of **2a** and **2b** (10 µM, CH_3_CN/H_2_O 8/2, v/v) in the absence and presence (1:1 molar ratio) of different metal perchlorates and an excess of NaOH (10:1 molar ratio, pH ∼ 7.3).

Ligands **2a-b** were used for in‐situ metal recognition studies in aqueous solvent mixtures. A preliminary investigation was performed to gain insight into their coordination behavior, including coordination geometry, selectivity, and potential for sensing processes. The screening of the absorption spectra of **2a** and **2b** against the perchlorate salts of various metal ions (Zn^+2^, Hg^+^, Cu^+2^, Cd^+2^, Pb^+2^, and Ni^+2^) was performed in CH_3_CN/H_2_O (8:2, v/v) in the absence (pH between 5.5 and 6.4) and the presence of NaOH (pH between 7.3 and 7.8) (Figure 4 and Figure S3). Perchlorate salts were selected due to their weakly coordinating anion character.

The absorption spectra of both **2a** and **2b** (10 μM, CH_3_CN/H_2_O 8/2, v/v, Figure 4) were characterized, in the region analyzed, by the presence of an intense band centered at 260 nm that must involve aromatic *π* − *π* ^∗^ electronic transitions of the aromatic unit of the Schiff base, with a shoulder visible in the low‐energy region of the band (275–300 nm, see Figures 4 and S3) that has been assigned to *π* − *π* ^∗^ electronic transitions of the imine C=N bond [78, 79]. Two additional, less intense, intramolecular charge transfer (ILCT) bands are usually found at lower energies and have been associated with the presence of protonated and deprotonated species with the participation of excited state intramolecular proton transfer (ESIPT) for the protonated enolamine tautomer [78, 79]. For ligands **2**, under neutral conditions, only a band centered at 325 nm is visible, suggesting its association with the ILCT of the protonated form. The addition of metal ions to **2a** and **2b** (1:1 molar ratio) led to the appearance of a broad band centered at around 375 nm for Zn^2+^ and Cu^2+^ complexes, which could be attributed to an ILCT of the deprotonated form, suggesting that complexation is accompanied by the deprotonation of the phenolic fragment. In the case of Cu^2+^, two intense new bands also appear at 240 and 275 nm that can be assigned to metal‐to‐ligand charge transfer (LMCT) processes. Upon the addition of an excess of NaOH (10:1 molar ratio), a second low‐intensity band is visible around 375 nm, particularly at higher concentrations (Figure S3), corresponding to the formation of the phenolate, and that seems to be more relevant for the phenylalanine derivative **2b** [79].

The addition of metal ions to **2a** and **2b**, in the presence of a base, shows the band centered at 375 nm for **2a**‐Cu^2+^, Ni^2+^, Pb^2+^ and Zn^2+^, and for all the metals tested in the case of **2b**. Furthermore, the appearance of an intense band at 275 nm is observed for Ni^2+^ complexes.

The addition of metal ions to a more concentrated solution of **2a** and **2b** (225 mM, CH_3_CN/H_2_O 8/2, v/v) in the absence of an added base displayed the band centered at 375 nm for **2a**‐Cu^2+^, Zn^2+^ and Ni^2+^ and for **2b**‐Cu^2+^, Zn^2+^ and Ni^2+^. This was accompanied by the appearance of a broad, small band centered at 400 nm for Hg^2+^, along with the observation of a clearly visible shoulder around 320 nm. In the presence of an excess of NaOH (2.25‐mM 10:1 molar ratio) the addition of metal ions to **2a** and **2b** (1:1 molar ratio) produced an increase in the intensity of the band centered at 370 nm along with a slight shift to a lower wavelength for the band at 320 nm, which became less visible for all the metals tested. Furthermore, the presence of a new band at 550 nm is observed for Cu^2+^ complexes suggesting the involvement of square planar to square pyramidal coordination geometries (ESI, Figure S3). Such a band has been reported for other Cu^2+^ complexes with *C*_2_‐symmetric pseudopeptidic ligands, whose crystal structures displayed square planar copper coordination [35, 39].

**FIGURE 5:**
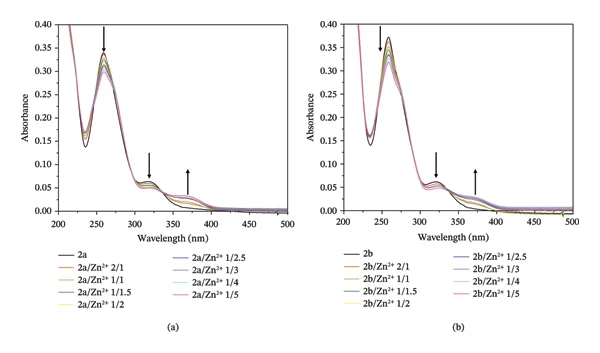
(a) UV–Visible spectra of **2a** (10 µM) after adding various aliquots of Zn^+2^ (1 mM, final molar ratios indicated in the inset) in CH_3_CN/H_2_O (8/2, v/v) solution. (b) UV–Visible spectra of **2b** (10 µM) after adding various aliquots of Zn^+2^ (1 mM, final molar ratios indicated in the inset) in CH_3_CN/H_2_O (8/2, v/v) solution.

To gain a better insight into the coordination behavior of **2a** and **2b** with Zn^2+^, titration studies were performed with an increasing concentration of Zn^2+^(1 mM). The corresponding absorption spectra for **2a** and **2b** showed a gradual decrease at the 320 nm band accompanied by an increase in the broad band intensity at 375 nm, with the presence of an isosbestic point at around 336 nm (Figure 5). A gradual decrease of the absorption band at 259 nm was also evident with the increase in the concentration of zinc ions. These data agree well with the suggested deprotonation of the Schiff base upon complexation.

**FIGURE 6:**
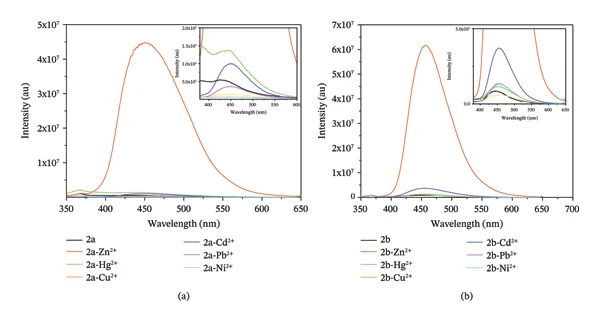
Fluorescence spectra of **2a** and **2b** (5 µM) after adding various metal perchlorates (2.8 mM, 1.8:1 ligand:metal molar ratio) in CH_3_CN/H_2_O (8/2, v/v) solution (λ_ex_ = 332 nm).

On the other hand, the fluorescence emission spectrum of **2a** undergoes a pronounced transformation upon excitation at 332 nm in the presence of a substoichiometric amount of Zn^2+^ (**2a**:Zn^2+^ molar ratio of 1.8:1). Under these conditions, an approximately 300% enhancement in emission intensity is observed, giving rise to a strong band centered at ∼450 nm on the addition of Zn^2+^ (Figure 6a), whereas the free ligand exhibits only weak basal fluorescence centered at ∼ 430 nm. An analogous behavior was observed for ligand **2b** (Figure 6b). By comparison, the other metal ions evaluated (Hg^2+^, Cu^2+^, Cd^2+^, Pb^2+^, and Ni^2+^) induce only minor perturbations in the basal emission, which remain negligible relative to the pronounced “turn‐on” response elicited by Zn^2+^ (see the enlarged insets in Figure 6a, b). Specifically, Cd^2+^ produce slight increases in fluorescence intensity, while coordination with Cu^2+^ results in modest quenching of the ligand’s intrinsic emission.

Fluorescence emission titration experiments for the **2a**–Zn^2+^ system, conducted in the same water/acetonitrile medium, reveal a progressive increase in emission intensity upon incremental addition of Zn^2+^, without any significant spectral shift in the emission maximum (Figure S4).

Given the marked and selective fluorescence enhancement observed for Zn^2+^ in isolated systems, the potential application of ligands **2a** and **2b** as fluorescent probes for Zn^2+^ detection was further explored. However, significant interference effects were detected in multimetal environments. When experiments were performed using equimolar mixtures of M^2+^ ions (Zn^2+^, Hg^2+^, Cu^2+^, Cd^2+^, Pb^2+^, and Ni^2+^; 0.1 mM each), a substantial decrease (∼90%) in emission intensity was observed, even at ligand concentrations as high as 0.7 mM (Figure S5, ESI). A detailed stoichiometric and thermodynamic analysis indicates that this behavior is primarily governed by competitive binding equilibria rather than direct quenching of the emissive Zn complex. In the equimolar mixture, the total concentration of competing metal ions reaches 0.5 mM (total metal pool of 0.6 mM). As supported by potentiometric data (Table 2), Cu^2+^ exhibit higher cumulative stability constants with the ligand than Zn^2+^. This thermodynamic preference, consistent with the Irving–Williams series, results in preferential sequestration of the ligand by this competing metal ion. The resulting complexes are largely nonemissive, primarily due to paramagnetic quenching effects associated with ions such as Cu^2+^ and Ni^2+^.

Consequently, despite an apparent excess of ligand relative to Zn^2+^, the effective concentration of free ligand available for Zn^2+^ coordination is significantly diminished. Furthermore, independent titration studies (Figure S4, ESI) demonstrate that maximal fluorescence output intrinsically requires a substantial excess of Zn^2+^. Only when the ligand concentration exceeds the binding capacity of the competing metal ions does Zn^2+^ complexation become significant, leading to a gradual recovery of fluorescence.

Although this competitive binding phenomenon currently limits the applicability of the system as a selective sensor in complex matrices, it provides valuable insights for future optimization. In particular, the incorporation of selective masking agents to sequester interfering transition metal ions represents a promising strategy to enhance Zn^2+^ detection under realistic analytical conditions [80].

**FIGURE 7:**
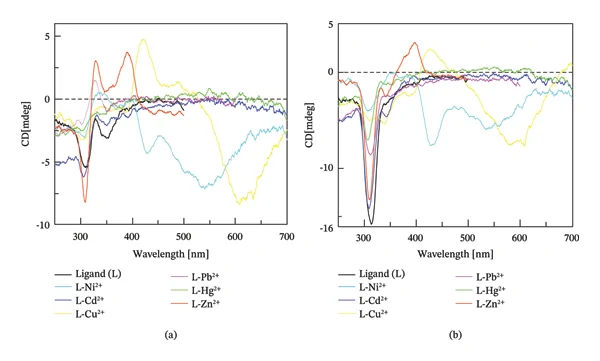
Partial CD spectra of **2a** (225 µM) (left) and **2b** (right) (225 µM) in the presence of base (2.25 mM) after adding various metal perchlorates (225 µM) in CH_3_CN/H_2_O (8/2 v/v). Final pH ∼ 7.8.

The presence of chiral amino acid fragments in compounds **2a** and **2b** allows performing CD measurements. Thus, the complexation of **2a-b** with the different metal ions was also studied by CD in the presence of an excess of NaOH (Figure 7). Changes in the CD spectral pattern upon addition of M^2+^ can be used to identify complex formation. It must be noted that changes in the intensity of the bands (molar ellipticity) are often associated to the degree of chiral ordering. Compounds **2a** and **2b** in CH_3_CN/H_2_O (8:2 v/v, 225 μM) in the presence of an excess of NaOH (10:1 molar ratio NaOH:ligand) displayed a negative CD signal (Δε = −0.76 and −2.25, respectively) at *ca.* 315 nm. A second much‐less intense negative signal is also observed around 350 nm. Such signals are related to the π–π∗ and ILCT transitions from the Schiff‐base fragment, indicating a high degree of chiral ordering, most likely associated to the existence of a strong network of hydrogen bonding with the participation of imine, phenol/phenoxide, and amide fragments. Interestingly, the higher intensity of the CD signal at 315 nm for **2b** suggests that the benzylic side chain in this compound favors the presence of more rigid conformations, decreasing conformational flexibility. The addition of an equimolar amount of the different M^2+^ to **2a** and **2b** is accompanied, in most instances, by the decrease in intensity of these negative CD signals at *ca.* 315 and 350 nm, suggesting that complexation reduces the proximity of the Schiff‐base moiety to the chiral center. Furthermore, in the presence of Cu^2+^, a split‐Cotton effect (−,+) was observed with both ligands, displaying the minimum around 600 nm and the maximum at *ca.* 430 nm and passing through zero in the region close to 500 nm. This signal is in line with the absorption detected in the UV–Vis spectrum and similar to the one observed with other *C*_2_‐symmetric pseudopeptides, confirming the presence of a square planar complex [39]. Interestingly, for Ni^2+^, two negative bands centered at 430 and 550 nm are present. Though square planar NiL complexes have always been observed with other pseudopeptidic systems [38, 39], a similar CD pattern has been reported for some pseudooctahedral Ni^2+^ complexes [81, 82], which agrees well with the higher number of donor atoms at each of the coordination subunits in **2**. Rather surprisingly, the addition of Zn^2+^ is accompanied by the appearance of a positive CD signal at 392 nm that must be related to the LMCT transition band observed in UV. Absorption and CD spectra of the zinc complexes should be featureless in this region, as expected for a d^10^ metal. This suggests a significant conformational change in the Schiff‐base unit upon coordination to Zn^2+^. No significant CD pattern changes were detected upon the addition of Hg^2+^, Cd^2+^, or Pb^2+^. To have a better insight into the CD behavior, titration studies were performed for **2b** in CH_3_CN/H_2_O (8:2 v/v, 225 mM) in the presence of NaOH (molar ratio NaOH:**2b** 10:1), with increasing concentrations of Cu^2+^, Zn^2+^, and Ni^2+^ (Figure S6–S8). The copper‐titrated absorption spectra showed a gradual decrease for the negative CD band at 314 nm until disappearance for a 1:1 molar ratio ligand: metal, accompanied with an increase in the broad band intensities for the positive and negative CD bands at 325 and 590 nm, respectively (Figure S6). The nickel‐titrated absorption spectra for **2b** showed a gradual decrease at the negative CD band at 314 nm until disappearance for a 1:3 molar ratio ligand: metal, accompanied with a small increase in the broad negative band intensity at 429 nm (Figure S7). Finally, for zinc‐titrated spectra, the gradual decrease of the negative CD band at *ca.* 310 nm upon metal addition is observed accompanied with an increase in the positive CD signal at 396 nm (Figure S8).

### 3.4. X‐Ray Diffraction for the Zn_2_L_2_ Complex of 2a

Synthesis of the corresponding Zn(II) complexes was attempted by reacting ligand **2a** with ZnCl_2_ in MeOH/CHCl_3_ (1:1) in the presence of Et_3_N (Scheme 2). In the case of **3a**, a high‐purity complex was obtained and fully characterized by ^1^H NMR, ^13^C NMR, FT‐IR, and MS (see ESI Figures S15–17). In contrast, applying the same conditions to ligand **2b** afforded a product displaying a highly complex ^1^H NMR spectrum, consistent with the presence of multiple species; consequently, complex **3b** was not isolated and no further purification or recrystallization was pursued.

**FIGURE 8:**
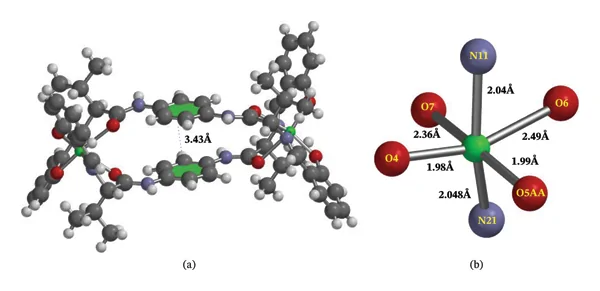
Solid‐state structure of **3a**. (a) Structure showing 2:2 ligand/metal stoichiometry. (b) Ligand‐zinc distances of the distorted square bipyramidal geometry.

Attempts to obtain diffraction‐quality crystals from complex **3a** prepared under the conditions described above were unsuccessful. However, single crystals suitable for X‐ray analysis were obtained by slow evaporation of an acetonitrile solution containing ligand **2a** and Zn(OAc)_2_, which afforded crystalline **3a**. The solid‐state structure of **3a** provided unambiguous evidence for the bimetallic complex formation between ligand **2a** and Zn^2+^ (Figure 8, see ESI for crystallographic data Tables S1–S7 and SCXDR CIF file as Supporting Information). The crystal lattice reveals the formation of a discrete entity involving two ligands and two Zn^2+^ metal ions, resulting in a molecular box like arrangement. Each zinc ion was found to be strongly coordinated to two imine nitrogen atoms and two phenolate oxygen atoms of two different ligands, as well as to two carbonyl oxygen atoms of the contiguous peptide groups. This arrangement leads to a distorted square bipyramidal geometry around the zinc ion, with the imine nitrogen atoms occupying the apical positions while the oxygen atoms of the pseudopeptide and phenolate moieties complete the equatorial coordination plane of the Zn metal. The Zn‐nitrogen (imine) bond distances were found to be 2.04 Å, whereas the Zn–O (phenolic) and Zn–O (peptidylic) bond distances are found to be 2.0 and 2.4 Å, respectively. Thus, the Schiff‐base moiety provides a stronger contribution to the coordination to the metal ion than the pseudopeptidic fragment. In fact, deprotonation of the N–H group of the amide is not observed, in contrast to the observation with other C_2_‐symmetric pseudopeptides lacking the Schiff‐base component.

The angle formed between the two apical nitrogen atoms with the zinc and the two phenolic oxygen atoms from the two different ligands with that of zinc ion were found to be 157° and 76°, respectively. The inwardly tilted and strained bond angle resulting in the formation of a virtual boxed shaped arrangement is further stabilized by the intermolecular stacking interaction between the aromatic spacer moieties. The distance between the centroid of the stacked aromatic benzene ring is found to be 3.43 Å. Interestingly, the two asymmetric units are connected through strong hydrogen bonding interaction between the amidic hydrogen with water molecule with a bond distance of 2.0‐Å solvent acetonitrile molecule was also found to be involved in hydrogen bonding interaction with the amide hydrogen with a bond distance of 2.2 Å.

The observed coordination behavior contains some similarities with other reported structures for other Zn‐pseudopeptide complexes [37, 38, 40]. In most instances, octahedral coordination geometries are observed [37, 40], with only a few complexes displaying square planar–pyramidal arrangements of the donor atoms [37, 38]. In contrast to the parent ligand **1**, which yields coordination polymers through the coordination of each metal center to two or three ligands, yielding 1D, 2D, or 3D coordination polymeric networks (metal:ligand ratios 1:1, 1:1.33, and 1:1.5) [37, 38], a discrete 2:2 metal–ligand complex is observed for **2a** in line with the design approach to ligands **2**. For **3a**, coordination takes between the oxygen atom of the carbonyl group with a distance of 2.4 Å, which is larger than the 2.14–2.23‐Å distances observed for related pseudopeptidic octahedral metal complexes [39, 40]. An even shorter (2.0 Å) has been reported for a square‐pyramidal complex from a ligand with an aliphatic spacer, but is this case a deprotonated amide group is involved [38]. In contrast, the distance between the deprotonated phenolic oxygen atom and the metal is 1.98 Å, shorter than the 2.06 Å distance observed a related pseudopeptidic metal complex [40].

## 4. Conclusions

In summary, we have designed and synthesized two new multifunctional open‐chain ligands (**2a** and **2b**) based on C2‐symmetric pseudopeptidic scaffolds incorporating valine or phenylalanine subunits and terminal salicylaldimine (Schiff base) fragments. The central advance of this work is the programmed formation of discrete M_2_L_2_ metallosupramolecular architectures from flexible pseudopeptidic ligands, a coordination outcome that is particularly favored for Zn(II). This behavior arises from a deliberate combination of design vectors: (i) a rigid *p*‐phenylene spacer that enforces subunit disposition and (ii) multidentate, multifunctional donor sets within each salicylaldimine subunit (phenolate/phenol O, imine N, and amide O, or the amide N upon deprotonation). By providing a sufficiently rich donor complement within the M_2_L_2_ framework, these ligands minimize the need for additional donor atoms from other strands, thereby biasing assembly toward dinuclear, discrete species rather than extended coordination polymers. Steric contributions from the salicylaldimine aromatic ring and the amino‐acid side chains are also expected to support this discrete topology.

The coordination behavior toward Zn(II) and Cu(II) was established by a combined potentiometric and spectroscopic analysis, which consistently indicates the formation of binuclear 2:2 complexes, which was further confirmed by single crystal X‐ray diffraction studies for the Zn^2+^ complex **3a**. The solid‐state structure reveals a distorted square‐bipyramidal Zn(II) environment built from two ligands, with imine nitrogen donors and amide/phenolate oxygen atoms completing the coordination sphere in the targeted dimeric arrangement. Notably, despite the conformational flexibility of ligands **2**, M_2_L_2_ assembly affords high stability constants (log *K* ≈ 15–23). The comparatively small Cu(II)/Zn(II) stability differences—especially for the valine derivative—further support that the present design is particularly well matched to Zn(II) coordination.

In line with this strong Zn(II) binding, complex formation produces a pronounced fluorescence turn‐on (> 300%), highlighting the potential of this ligand family as a platform for Zn(II)‐responsive emitters, although interference from other metal ions limits selectivity in its current form. Finally, the intrinsic chirality of **2a-b** enables CD spectroscopy as an additional, highly sensitive probe of complexation through diagnostic changes in the Schiff‐base region (especially informative for Zn(II)) and the emergence of intense d–d features for Ni(II) and Cu(II).

## Author Contributions

Arpna Tamrakar: investigation, formal analysis, and data curation. Mrituanjay D. Pandey: writing–review and editing, investigation, formal analysis, data curation, and conceptualization. Armando Ferrer, Vicente Martí‐Centelles, Santiago V. Luis, and Belén Altava: writing–review and editing, writing–original draft, visualization, validation, supervision, resources, project administration, methodology, funding acquisition, formal analysis, and conceptualization. Eduardo García‐Verdugo: visualization, validation, supervision, resources, project administration, funding acquisition, and conceptualization.

## Funding

This research was funded by Ministerio de Ciencia e Innovación/Agencia Estatal de Investigación, MCIN/AEI/10.13039/501100011033, and ERDF A way of making Europe, grant numbers PDI2021‐124695OB‐C22 and PID2024‐159264OB‐C21. V.M.‐C thanks the financial support from Generalitat Valenciana (APOSTD/2013/041, CIDEGENT/2020/031, and ESGENT/2024/001) and from project CNS2023‐144879 funded by MICIU/AEI/10.13039/501100011033 and European Union NextGenerationEU/PRTR. A.F.S. thanks the financial support from Projecte de cooperació internacional al desenvolupament de la Universitat Jaume I (PCoopUJI‐20‐07).

## Conflicts of Interest

The authors declare no conflicts of interest.

## Supporting Information

Additional supporting information can be found online in the Supporting Information section.

## Supporting information


**Supporting Information 1** Additional supporting information can be found online. Supporting Information (PDF file) containing characterization including crystallographic details, UV–Vis, CD, NMR, MS, and fluorescence spectra.

**Supporting Information 2** X‐ray crystallography (CIF file): Crystal structure of complex **3a** was deposited at the Cambridge Crystallographic Data Centre (CCDC) with deposition number 2478306. These data can be obtained free of charge from the Cambridge Crystallographic Data Centre via https://www.ccdc.cam.ac.uk/data_request/cif.

## Data Availability

Data used to support the findings of this study is included in the manuscript and in the supporting information.
