# Supplementary material for: C 2‐Symmetric Pseudopeptidic Schiff Bases Drive Discrete M2L2 Complexes: Coordination, Stability, and Photophysical Response
Source: Bioinorg Chem Appl. 2026 Sep 23;2026:1063983. doi: 10.1155/bca/1063983 (PMC13601859; doi:10.1155/bca/1063983)
Supplement: Supplementary file 1 [file BCA-2026-1063983-s002.pdf]

ELECTRONIC SUPPORTING INFORMATION

C_2-Symmetric Pseudopeptidic Schiff Bases Drive Discrete M_2L_2 Complexes: Coordination, Stability, and Photophysical Response

Mrituanjay D. Pandey,[1,2,] Arpna Tamrakar,[1] Vicente Martí-Centelles,[2,3,5] Armando Ferrer,[2,4,6]* Eduardo. García-Verdugo,[2] Santiago V. Luis, [2,]* Belén Altava,[2,]**

[1]Department of Chemistry, Institute of Science, Banaras Hindu University Varanasi–221005, India.

[2]Universitat Jaume I, Departamento de Química Inorgánica y Orgánica, Av. Sos Baynat, s/n, E-12071 Castellón, Spain.

[3]Instituto Interuniversitario de Investigación de Reconocimiento Molecular y Desarrollo Tecnológico (IDM), Universitat Politècnica de València, Universitat de València, Camino de Vera, s/n., Valencia 46022, Spain.

[4]Departamento de Orgânica, Instituto de Química, Universidade Federal da Bahía, Rua Barão de Jeremoabo, 147 – Campus Universitário de Ondina, 40170-115, Salvador, Bahía, Brazil.

[5]Departamento de Química, Universitat Politècnica de València, Camí de Vera s/n, 46022 Valencia, Spain

[6]Departamento de Química, Facultad de Ciencias Naturales y Exactas, Universidad de Oriente, Av. Patricio Lumumba s/n, 90200, Santiago de Cuba, Cuba.

Content:

Figure S1. ^{1}H NMR spectra of **2a** before and after acidic treatment

Figure S2. Conformation energy diagram for tautomeric forms of ligand **2a**

Figure S3. UV-Visible spectra of **2a** and **2b** in the presence of M(II)

Figure S4. Fluorescence spectra of **2a** in the presence of Zn(II)

Figure S5. Fluorescence spectra of **2a** in the presence of a mixture of equimolar amount of M^{2+} ions

Figure S6. CD spectra of **2b** in the presence of base after adding different amounts of Cu^{2+}

Figure S7. CD spectra of **2b** in the presence of base after adding different amounts of Ni^{2+}

Figure S8. CD spectra of **2b** in the presence of base after adding different amounts of Zn^{2+}

Crystalographic data of **3a**

Figure S9. Partial ^{1}H NMR spectrum for **2a**

Figure S10. Partial ^{13}C NMR spectrum for **2a**

Figure S11. ESI-MS (+ -) spectra for **2a**

Figure S12. Partial ^{1}H NMR spectrum for **2b**

Figure S13. Partial ^{13}C NMR spectrum for **2b**

Figure S14. ESI-MS (+) spectra for **2b**

Figure S15. Partial ^{1}H NMR spectrum for **3a**

Figure S16. Partial ^{13}C NMR spectrum for **3a**

Figure S17. HRMS spectra for **3a**

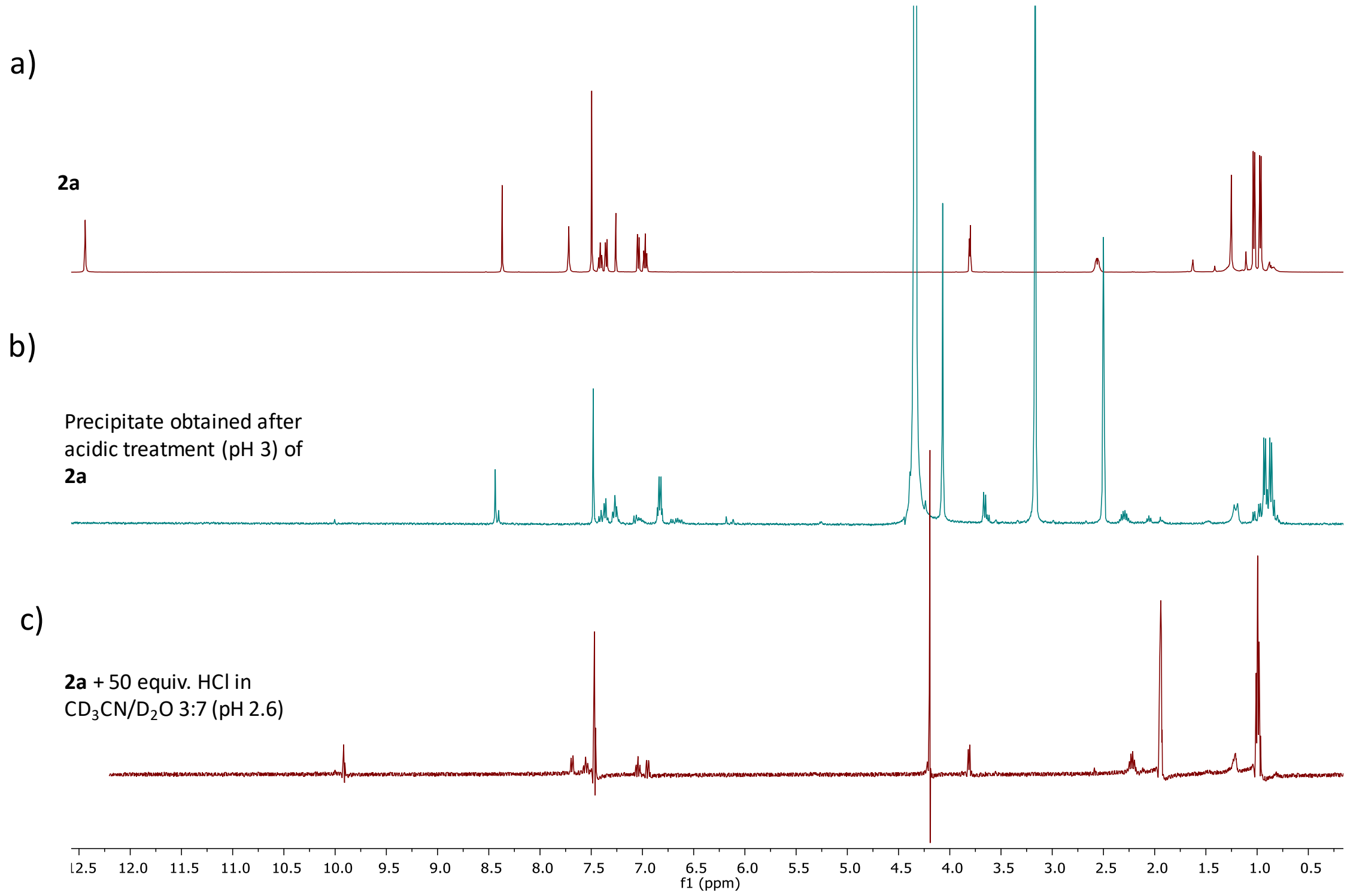

Figure S1. ^1H NMR spectra of **2a** before and after acidic treatment with HCl. a) **2a** (6 mM in $CDCl_3$). b) **2a** (4 mM, CD_3OD/DMSO-d_6) after treatment with H_2O/CH_3CN 7/3 pH 3. c) **2a** (3 mM in D_2O/CD_3CN 7/3 pH 2.6).

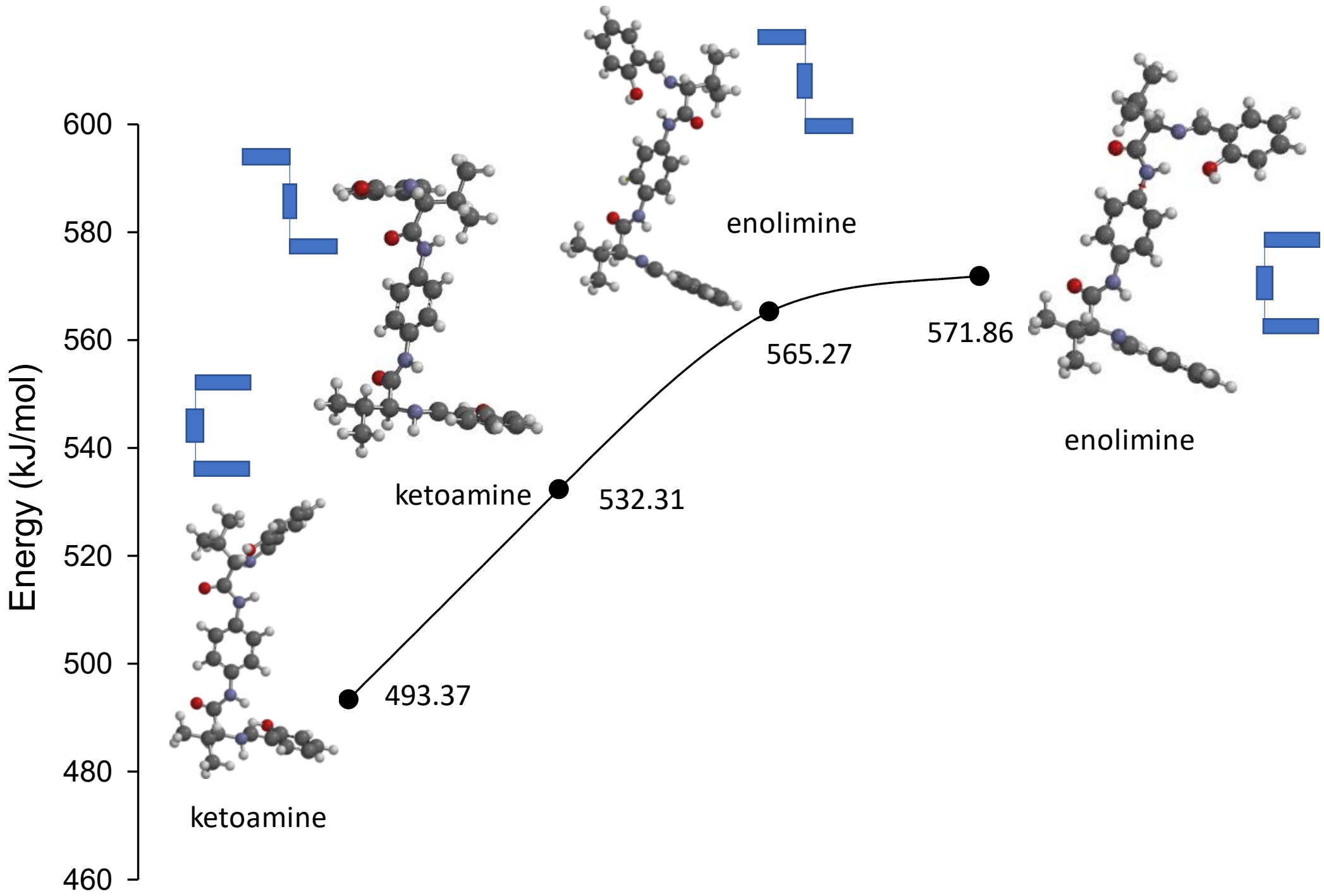

Figure S2. Conformation energy diagram for tautomeric forms of ligand **2a** calculated by Spartan 08 (minimization energy). Deppmeier, B. J.; Driessen, A. J.; Hehre, T. S.; Hehre, W. J.; Johnson, J. A.; Klunzinger, P. E.; Leonard, J. M.; Pham, I. N.; Pietro, W. J.; Jianguo, Y.Spartan'08, build 132 (Mar 27, 2009); Wavefunction, Inc.: Irvine, CA, 2009.

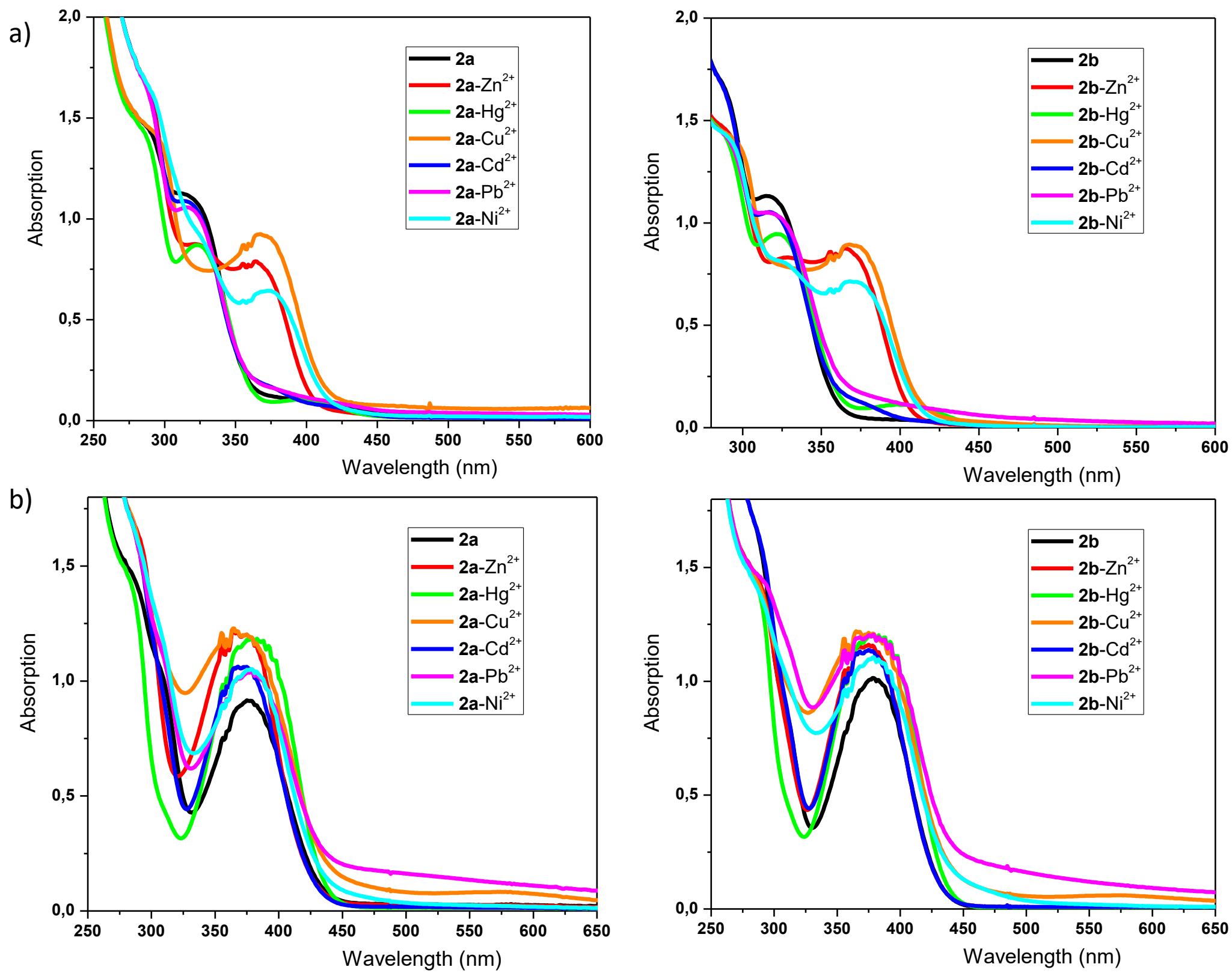

Figure S3. a) UV-Visible spectra of **2a** and **2b** (225 μM, H_2O/CH_3CN 2/8 v/v) in the presence of different metal perchlorates (1:1 molar ratio pH ~ 5.5). b) UV-Visible Spectra of **2a** and **2b** (225 μM, H_2O/CH_3CN, 2/8 v/v) in the presence of different metal perchlorates (1:1 molar ratio) and NaOH (0.1 M, 10:1 molar ratio pH ~7.8).

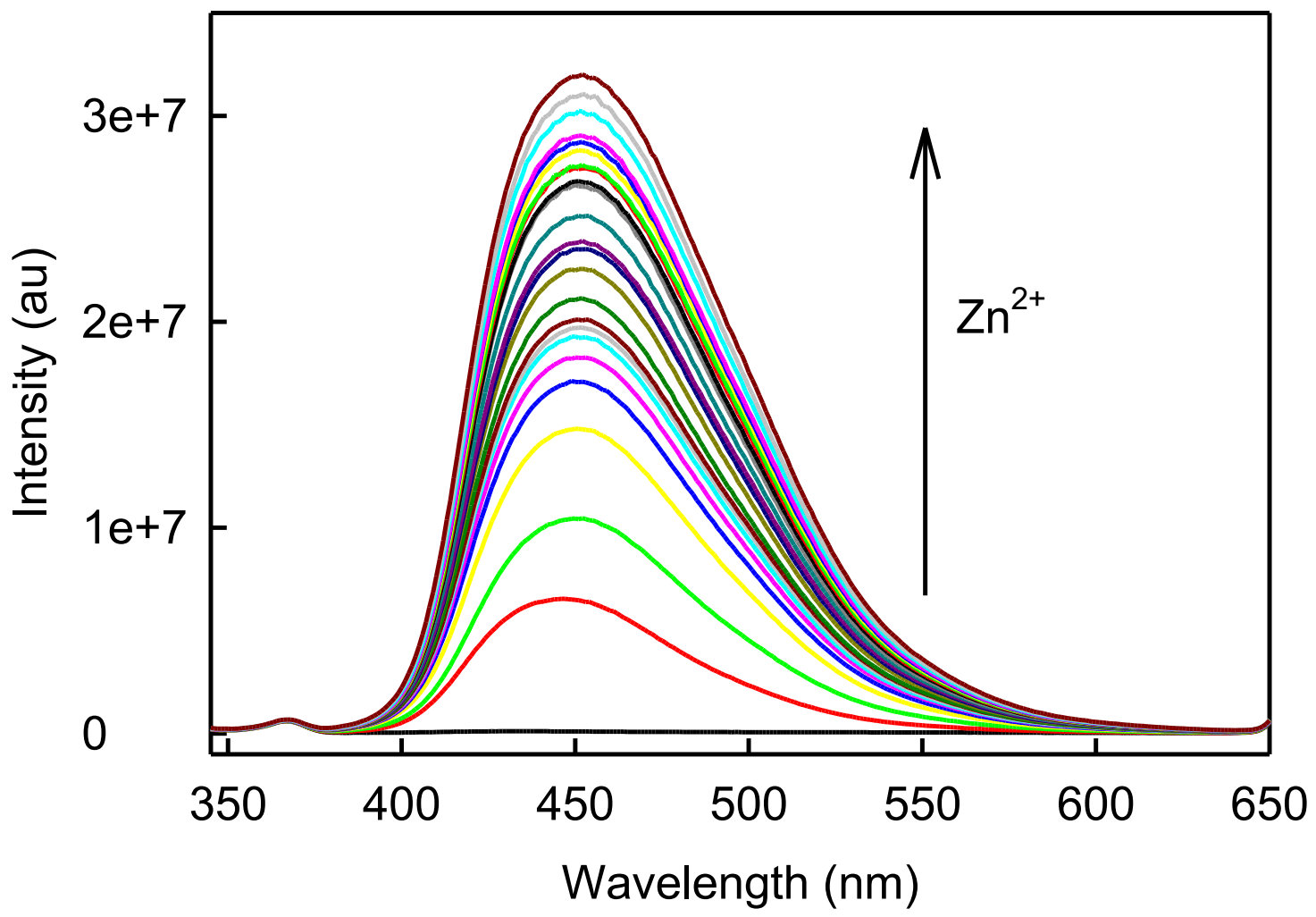

Figure S4: Fluorescence spectra of **2a** (5 µM) after adding various aliquots of Zn(II) (0 - 3 mM) in aqueous-acetonitrile solution (λ_{ex} = 332 nm).

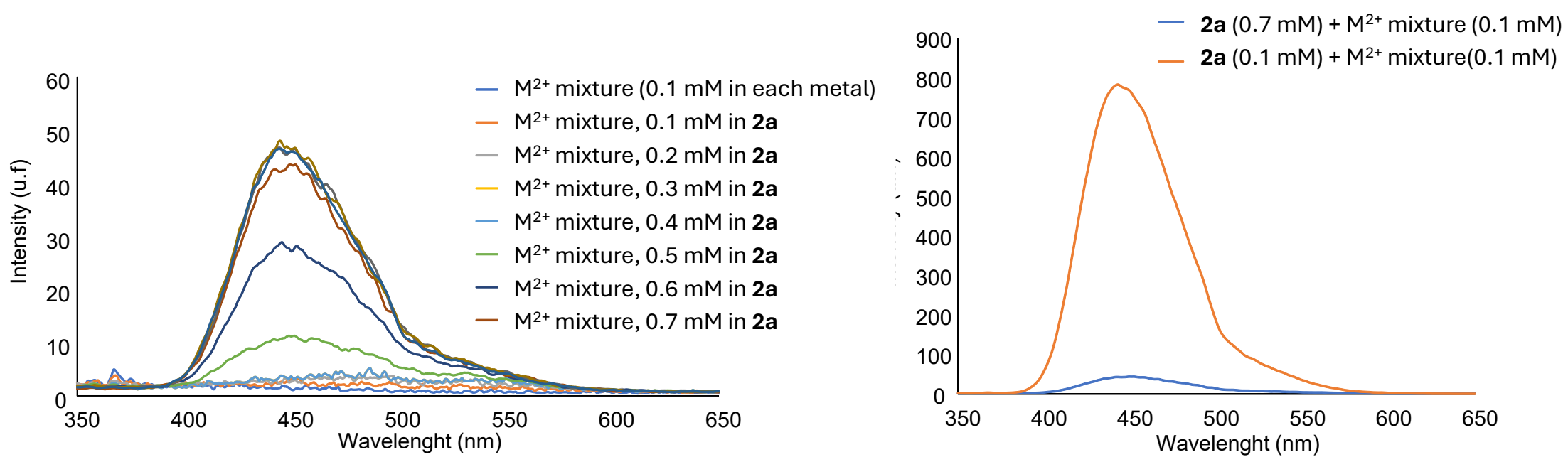

Figure S5. a) Fluorescence spectra of a mixture of equimolar amount of M^{2+} ions (0.1 mM) in the absence and presence of different amounts of ligand **2a** in water/acetonitrile, 2/8 v/v. b) Fluorescence spectra of **2a** in the presence of M^{2+}. λ_{ex} = 332 nm.

e

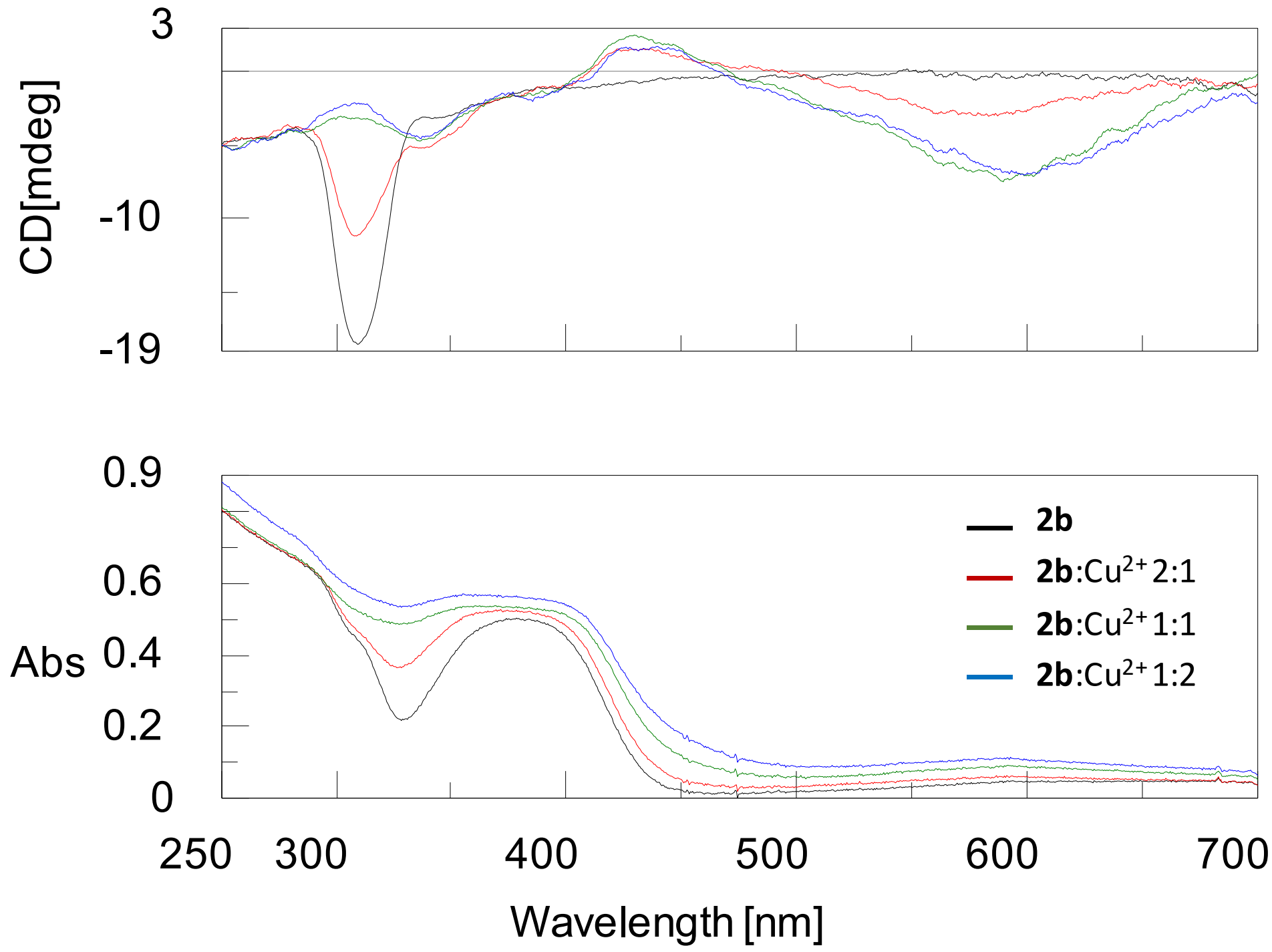

Figure S6. CD spectra of **2b** (225 µM) in the presence of base (10 moles / mol) after adding different amounts of Cu^{2+} (1 mM) in water/acetonitrile 2/8 v/v.

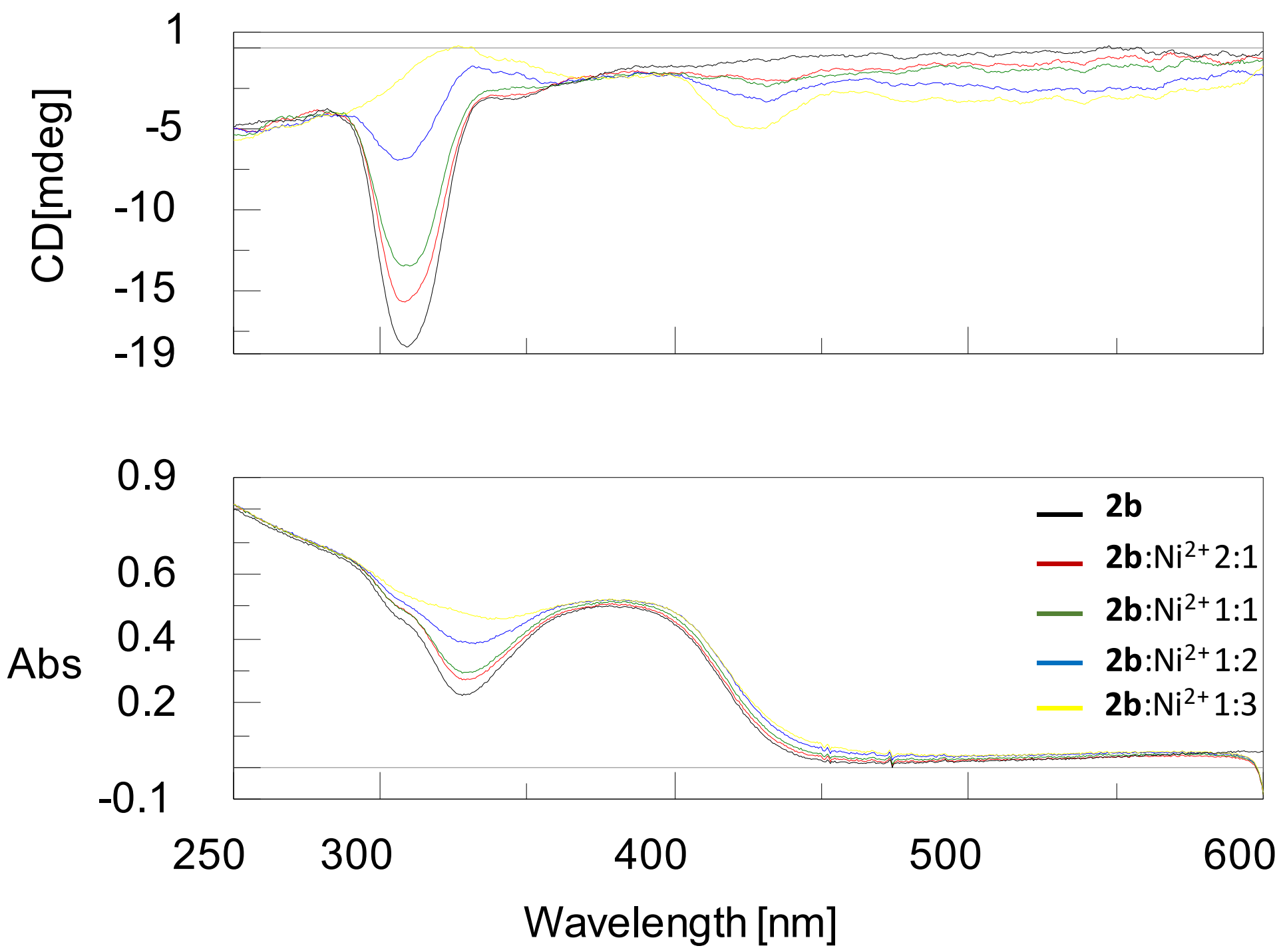

Figure S7. CD spectra of **2b** (225 μM) in the presence of base (10 moles / mol) after adding different amounts of Ni^{2+} (1 mM) in water/acetonitrile 2/8 v/v.

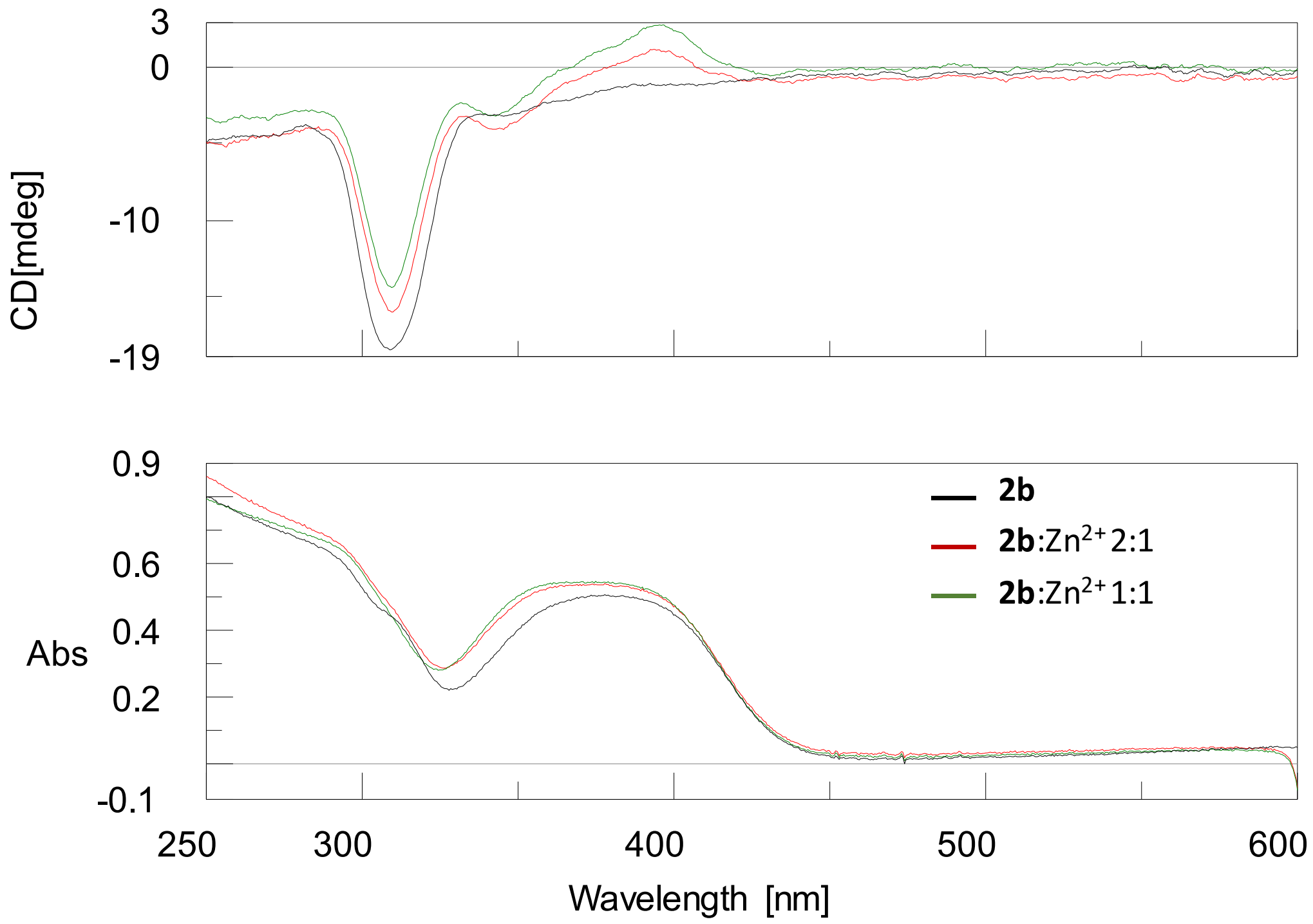

Figure S8. CD spectra of **2b** (225 μM) in the presence of base (10 moles / mol) after adding different amounts of Zn^{2+} (1 mM) in water/acetonitrile 2/8 v/v.

Crystalographic data

Table S1 Crystal data and structure refinement for 3a.

Identification code	3a
Empirical formula	$C_{64}H_{80}N_8O_{12}Zn_2$
Formula weight	1284.10
Temperature/K	293(2)
Crystal system	trigonal
Space group	$P3_121$
a/Å	18.9710(7)
b/Å	18.9710(7)
c/Å	28.1733(11)
α/°	90.00
β/°	90.00
γ/°	120.00
Volume/Å^3	8781.1(6)
Z	3
ρ_{calc}g/cm^3	0.728
μ/mm^{-1}	0.446
F(000)	2028.0
Crystal size/mm^3	0.8 × 0.6 × 0.2
Radiation	MoKα (λ = 0.71073)
2Θ range for data collection/°	6.1 to 58.8
Index ranges	-20 ≤ h ≤ 24, -26 ≤ k ≤ 24, -38 ≤ l ≤ 37
Reflections collected	63027
Independent reflections	14687 [R_{int} = 0.1156, R_{sigma} = 0.1538]
Data/restraints/parameters	14687/6/397
Goodness-of-fit on F^2	0.920
Final R indexes [I>=2σ (I)]	R_1 = 0.0923, wR_2 = 0.2307
Final R indexes [all data]	R_1 = 0.1995, wR_2 = 0.2629
Largest diff. peak/hole / e Å^{-3}	0.47/-0.27
Flack parameter	0.04(2)

Table S2 Fractional Atomic Coordinates (×10^4) and Equivalent Isotropic Displacement Parameters (Å^2×10^3) for 3a. U_{eq} is defined as 1/3 of of the trace of the orthogonalised U_{IJ} tensor.

Atom	*x*	*y*	*z*	U(eq)
C1	4448(4)	5269(4)	72.5(18)	87.1(16)
C1B	5640(4)	8315(4)	1995(2)	92.1(18)
C1D	3437(6)	5316(5)	2400(2)	132(3)
C1E	4256(4)	5627(4)	1653.5(18)	89.8(16)
C1F	6979(8)	8618(9)	2545(5)	181(5)
C1G	5799(5)	7725(5)	2146(3)	111(2)
C1M	5276(4)	6839(5)	2024(2)	98.1(18)
C1R	6447(7)	7887(8)	2425(3)	143(3)
C1S	6823(7)	9228(10)	2438(4)	184(5)
C1X	6446(6)	9455(6)	360(4)	155(3)
C2	4703(3)	5015(3)	464.6(16)	72.0(13)
C3	5113(4)	4614(4)	365.5(18)	93.2(18)
C4	5063(6)	9468(5)	495(3)	138(3)
C5	4256(4)	5564(4)	1114.9(17)	84.5(15)
C6	3498(4)	7333(5)	2655(2)	108(2)
C7	3723(5)	8995(5)	1658(3)	123(2)
C9	2999(6)	4298(5)	1731(3)	137(3)
C13	3411(5)	5203(4)	1844.3(19)	105.7(19)
C14	2674(5)	6819(4)	1158(2)	98.6(18)
C15	3646(5)	7553(4)	483(2)	100(2)
C17	2866(5)	7150(5)	685(2)	106.7(19)
C18	6164(5)	9086(6)	2122(3)	134(3)
C19	5565(5)	9037(5)	594(2)	120(2)
C444	6936(4)	6642(4)	-442(2)	91.1(17)
C445	6529(4)	7040(3)	-378.7(17)	78.5(15)
C446	6343(3)	7191(3)	58.7(18)	78.8(14)
C448	5538(4)	7695(4)	483.3(18)	87.4(16)
C449	5068(4)	8143(4)	409(2)	89.8(17)
N4	4593(3)	5157(3)	935.9(13)	73.7(11)
N11	4692(4)	6543(4)	1743.8(14)	94.0(15)
N21	4324(4)	7703(3)	673.9(17)	95.9(15)
N447	5919(3)	7604(3)	107.6(15)	81.4(12)
O1	4565(3)	9313(2)	1723.1(13)	95.3(11)
O4	4980(3)	8155(2)	1736.9(13)	93.1(11)
O5	2818(3)	6862(3)	2359.5(15)	113.7(14)
O5AA	3240(3)	6887(3)	1449.0(13)	93.5(11)
O6	3978(3)	5917(3)	872.7(13)	104.6(13)
O7	5578(3)	7465(3)	891.8(15)	101.8(12)
Zn1	4390.9(4)	7211.3(4)	1307.68(19)	82.7(2)
C0AA	1881(7)	6444(6)	1277(3)	144(3)
C1AA	2179(7)	7059(6)	399(3)	137(3)
C3AA	1267(6)	6348(8)	1005(4)	168(4)
C2AA	1431(9)	6634(9)	583(6)	197(5)

Table S3 Anisotropic Displacement Parameters (Å^2×10^3) for 3a. The Anisotropic displacement factor exponent takes the form: $-2\pi^2[h^2a^{*2}U_{11}+2hka^*b^*U_{12}+\ldots]$.

Atom	U_{11}	U_{22}	U_{33}	U_{23}	U_{13}	U_{12}
C1	114(5)	106(4)	61(3)	3(3)	8(3)	70(4)
C1B	88(4)	101(5)	86(4)	-19(3)	-14(3)	46(4)
C1D	190(8)	123(6)	91(4)	2(4)	25(5)	84(6)
C1E	122(5)	91(4)	69(3)	-3(3)	4(3)	63(4)
C1F	166(11)	152(10)	219(12)	20(9)	-28(9)	75(10)
C1G	116(5)	140(7)	95(4)	-5(4)	-17(4)	78(5)
C1M	122(5)	118(6)	74(4)	4(3)	4(3)	75(5)
C1R	142(8)	165(9)	126(7)	5(6)	-8(6)	78(7)
C1S	129(8)	222(14)	163(10)	-26(9)	-16(7)	58(9)
C1X	160(8)	115(6)	159(8)	11(6)	25(7)	46(6)
C2	97(4)	74(3)	56(3)	-4(2)	-1(2)	51(3)
C3	126(5)	123(5)	58(3)	-5(3)	-3(3)	82(5)
C4	192(9)	121(6)	118(6)	2(5)	2(5)	91(6)
C5	120(5)	91(4)	56(3)	8(3)	7(3)	62(4)
C6	114(5)	130(5)	77(4)	-17(4)	-13(3)	59(4)
C7	132(7)	145(6)	109(5)	-15(4)	3(5)	82(6)
C9	182(8)	128(6)	102(5)	7(5)	24(5)	78(6)
C13	137(6)	125(5)	66(3)	8(3)	16(4)	73(5)
C14	105(5)	120(5)	71(4)	-19(3)	-10(3)	56(4)
C15	141(6)	117(5)	68(3)	-13(3)	-13(4)	84(5)
C17	112(5)	135(5)	70(3)	-22(4)	-13(4)	60(4)
C18	123(6)	145(8)	122(6)	-5(5)	-17(5)	59(6)
C19	164(7)	129(6)	81(4)	0(4)	1(4)	83(6)
C444	107(5)	105(4)	62(3)	1(3)	-3(3)	52(4)
C445	103(4)	89(4)	54(3)	5(2)	-5(3)	56(3)
C446	81(3)	86(4)	61(3)	8(3)	6(2)	36(3)
C448	109(4)	96(4)	55(3)	-1(3)	-3(3)	50(4)
C449	119(5)	98(4)	65(3)	-4(3)	0(3)	64(4)
N4	102(3)	81(3)	51(2)	4.6(19)	6(2)	55(3)
N11	131(4)	135(5)	49(2)	10(3)	7(3)	91(4)
N21	130(5)	105(4)	72(3)	-1(3)	0(3)	73(4)
N447	103(3)	94(3)	56(2)	1(2)	1(2)	56(3)
O1	114(3)	102(3)	76(2)	-9.8(19)	-9(2)	58(3)
O4	123(3)	104(3)	72(2)	-2(2)	-2(2)	72(3)
O5	125(4)	128(4)	91(3)	-17(2)	-1(3)	66(3)
O5AA	117(3)	109(3)	65(2)	-4.6(19)	-3(2)	64(2)
O6	150(4)	138(4)	60(2)	5(2)	4(2)	97(3)
O7	134(4)	117(3)	70(3)	5(2)	11(2)	74(3)
Zn1	114.7(5)	97.1(5)	53.4(3)	-1.2(3)	-3.5(3)	65.7(4)
C0AA	142(8)	169(8)	107(6)	-18(5)	1(6)	68(7)
C1AA	161(8)	169(8)	89(5)	-14(5)	-17(5)	89(7)
C3AA	128(7)	237(12)	110(7)	-6(7)	-19(6)	71(8)
C2AA	180(8)	227(9)	195(9)	-7(7)	-42(7)	110(7)

Table S4 Bond Lengths for 3a.

Atom	Atom	Length/Å
C1	C2	1.386(7)
C1	C3[1]	1.342(7)
C1B	C1G	1.365(10)
C1B	C18	1.342(11)
C1B	O4	1.345(7)
C1D	C13	1.578(9)
C1E	C5	1.522(7)
C1E	C13	1.489(10)
C1E	N11	1.527(8)
C1F	C1R	1.287(13)
C1F	C1S	1.365(16)
C1G	C1M	1.503(11)
C1G	C1R	1.358(11)
C1M	N11	1.243(8)
C1S	C18	1.445(14)
C1X	C19	1.591(12)
C2	C3	1.361(8)
C2	N4	1.391(6)
C3	C1[1]	1.342(7)
C4	C19	1.560(11)
C5	N4	1.323(7)
C5	O6	1.243(6)
C6	O5	1.415(7)
C7	O1	1.408(8)
C9	C13	1.522(10)
C14	C17	1.440(9)
C14	O5AA	1.305(7)
C14	C0AA	1.347(11)
C15	C17	1.402(10)
C15	N21	1.287(8)
C17	C1AA	1.467(11)
C19	C449	1.560(10)
C444	C445	1.335(8)
C444	C446[1]	1.412(8)
C445	C446	1.352(7)
C446	C444[1]	1.412(8)
C446	N447	1.382(7)
C448	C449	1.524(8)
C448	N447	1.340(7)
C448	O7	1.247(6)
C449	N21	1.438(8)
N11	Zn1	2.042(5)
N21	Zn1	2.047(5)
O4	Zn1	1.978(4)
O5AA	Zn1	1.990(4)
O7	Zn1	2.365(4)
C0AA	C3AA	1.329(12)
C1AA	C2AA	1.339(15)
C3AA	C2AA	1.278(15)

[1]+Y,+X,-Z

Table S5 Bond Angles for 3a.

Atom	Atom	Atom	Angle/˚	Atom	Atom	Atom	Angle/˚
C3[1]	C1	C2	119.7(5)	C444	C445	C446	121.9(5)
C18	C1B	C1G	117.7(7)	C445	C446	C444[1]	115.6(5)
C18	C1B	O4	119.3(7)	C445	C446	N447	119.9(5)
O4	C1B	C1G	123.0(7)	N447	C446	C444[1]	124.4(5)
C5	C1E	N11	103.6(4)	N447	C448	C449	117.9(5)
C13	C1E	C5	111.1(5)	O7	C448	C449	118.5(5)
C13	C1E	N11	112.3(5)	O7	C448	N447	123.6(6)
C1R	C1F	C1S	118.0(13)	C448	C449	C19	110.8(5)
C1B	C1G	C1M	123.6(7)	N21	C449	C19	110.1(5)
C1R	C1G	C1B	122.7(9)	N21	C449	C448	106.4(5)
C1R	C1G	C1M	113.7(8)	C5	N4	C2	129.8(4)
N11	C1M	C1G	126.3(6)	C1E	N11	Zn1	116.5(4)
C1F	C1R	C1G	122.0(11)	C1M	N11	C1E	118.3(5)
C1F	C1S	C18	120.9(13)	C1M	N11	Zn1	124.4(5)
C1	C2	N4	125.5(5)	C15	N21	C449	119.5(5)
C3	C2	C1	115.3(5)	C15	N21	Zn1	123.1(5)
C3	C2	N4	119.1(4)	C449	N21	Zn1	116.8(4)
C1[1]	C3	C2	125.0(5)	C448	N447	C446	131.0(5)
N4	C5	C1E	116.4(5)	C1B	O4	Zn1	125.2(4)
O6	C5	C1E	119.2(5)	C14	O5AA	Zn1	128.7(4)
O6	C5	N4	124.3(4)	C448	O7	Zn1	108.1(4)
C1E	C13	C1D	109.4(6)	N11	Zn1	N21	155.24(19)
C1E	C13	C9	109.9(6)	N11	Zn1	O7	83.73(17)
C9	C13	C1D	109.0(6)	N21	Zn1	O7	75.24(19)
O5AA	C14	C17	121.5(7)	O4	Zn1	N11	89.3(2)
O5AA	C14	C0AA	122.8(7)	O4	Zn1	N21	104.89(19)
C0AA	C14	C17	115.7(7)	O4	Zn1	O5AA	101.17(17)
N21	C15	C17	128.0(6)	O4	Zn1	O7	93.97(17)
C14	C17	C1AA	116.2(7)	O5AA	Zn1	N11	106.98(19)
C15	C17	C14	125.5(7)	O5AA	Zn1	N21	90.4(2)
C15	C17	C1AA	118.3(7)	O5AA	Zn1	O7	161.42(15)
C1B	C18	C1S	117.8(10)	C3AA	C0AA	C14	126.8(9)
C4	C19	C1X	115.4(7)	C2AA	C1AA	C17	118.1(9)
C4	C19	C449	108.4(6)	C2AA	C3AA	C0AA	118.0(11)
C449	C19	C1X	108.5(6)	C3AA	C2AA	C1AA	125.1(12)
C445	C444	C446[1]	122.5(5)				

[1]+Y,+X,-Z

Table S6 Hydrogen Atom Coordinates (Å×10^4) and Isotropic Displacement Parameters (Å^2×10^3) for 3a.

Atom	*x*	*y*	*z*	U(eq)
H1	4050(40)	5460(40)	150(20)	104
H1DA	3781	5134	2538	199
H1DB	2897	5001	2527	199
H1DC	3648	5880	2474	199
H1E	4568	5394	1793	108
H1F	7457	8727	2699	217
H1M	5402	6478	2172	118
H1R	6507	7457	2533	172
H1S	7144	9742	2570	221
H1XA	6745	9212	487	232
H1XB	6729	10026	430	232
H1XC	6395	9377	22	232
H3	5299	4441	621	112
H4A	4837	9334	181	207
H4B	5413	10047	520	207
H4C	4632	9290	723	207
H6A	3972	7354	2521	162
H6B	3398	7090	2965	162
H6C	3583	7875	2681	162
H7A	3634	9375	1480	184
H7B	3462	8902	1961	184
H7C	3500	8490	1487	184
H9A	3354	4094	1816	206
H9B	2882	4217	1397	206
H9C	2501	4013	1907	206
H13	3101	5434	1701	127
H15	3678	7735	173	120
H18	6105	9515	2011	160
H19	5628	9019	938	144
H444	7057	6558	-749	109
H445	6368	7217	-643	94
H449	4944	8143	71	108
H4	4770	4948	1142	88
H447	5895	7843	-146	98
H1A	4688	8974	1640	143
H5	2873	6507	2229	171
H0AA	1748	6230	1582	172
H1AA	2262	7289	98	164
H3AA	735	6081	1117	201
H2AA	995	6540	389	236

Table S7 Solvent masks information for 3a.

Number	X	Y	Z	Volume	Electron count	Content
1	-0.006	-0.005	-0.003	4635	850	

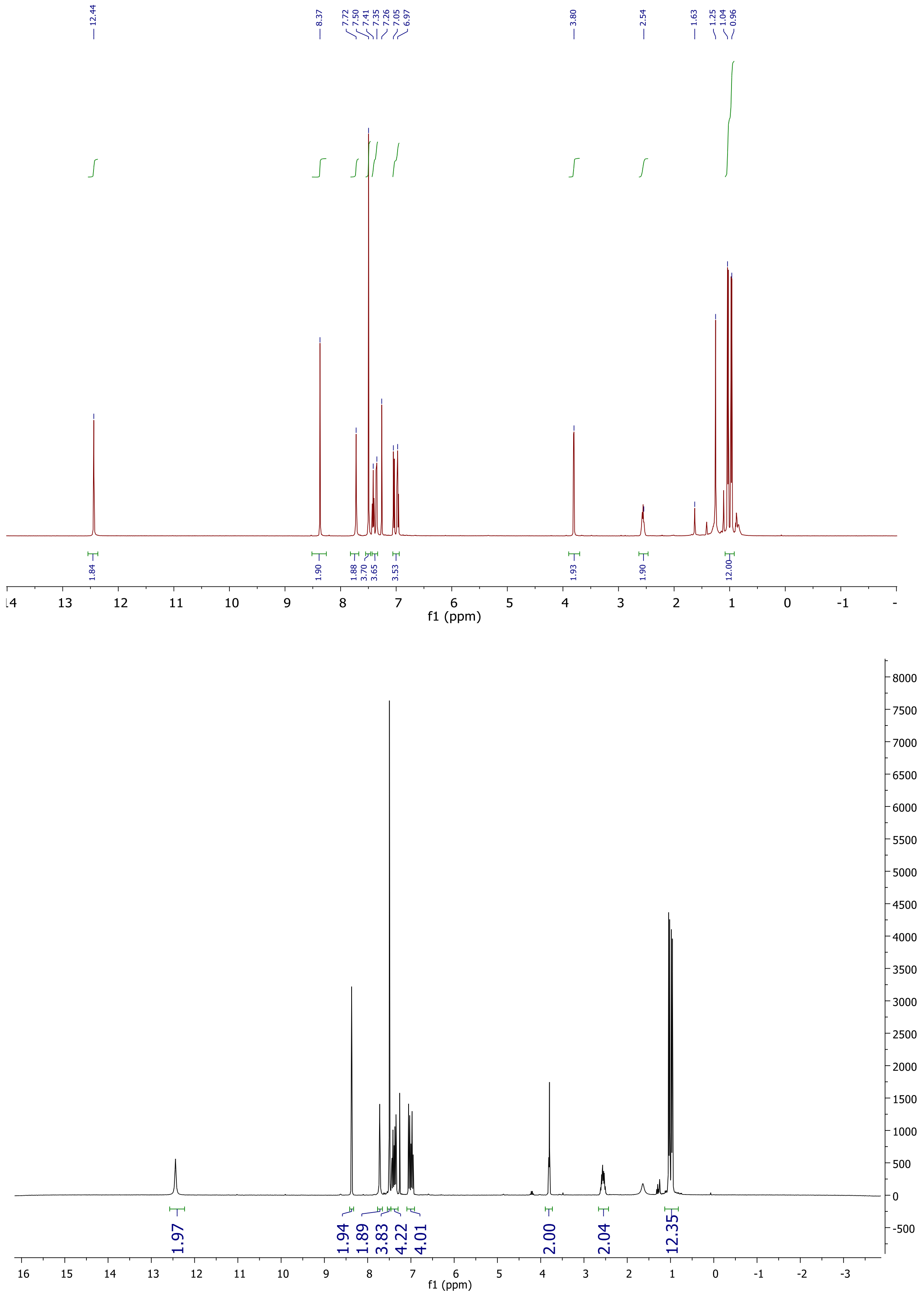

Figure S9. ^{1}H NMR spectrum for **2a** (300 MHz, $CDCl_3$, 5 mM)

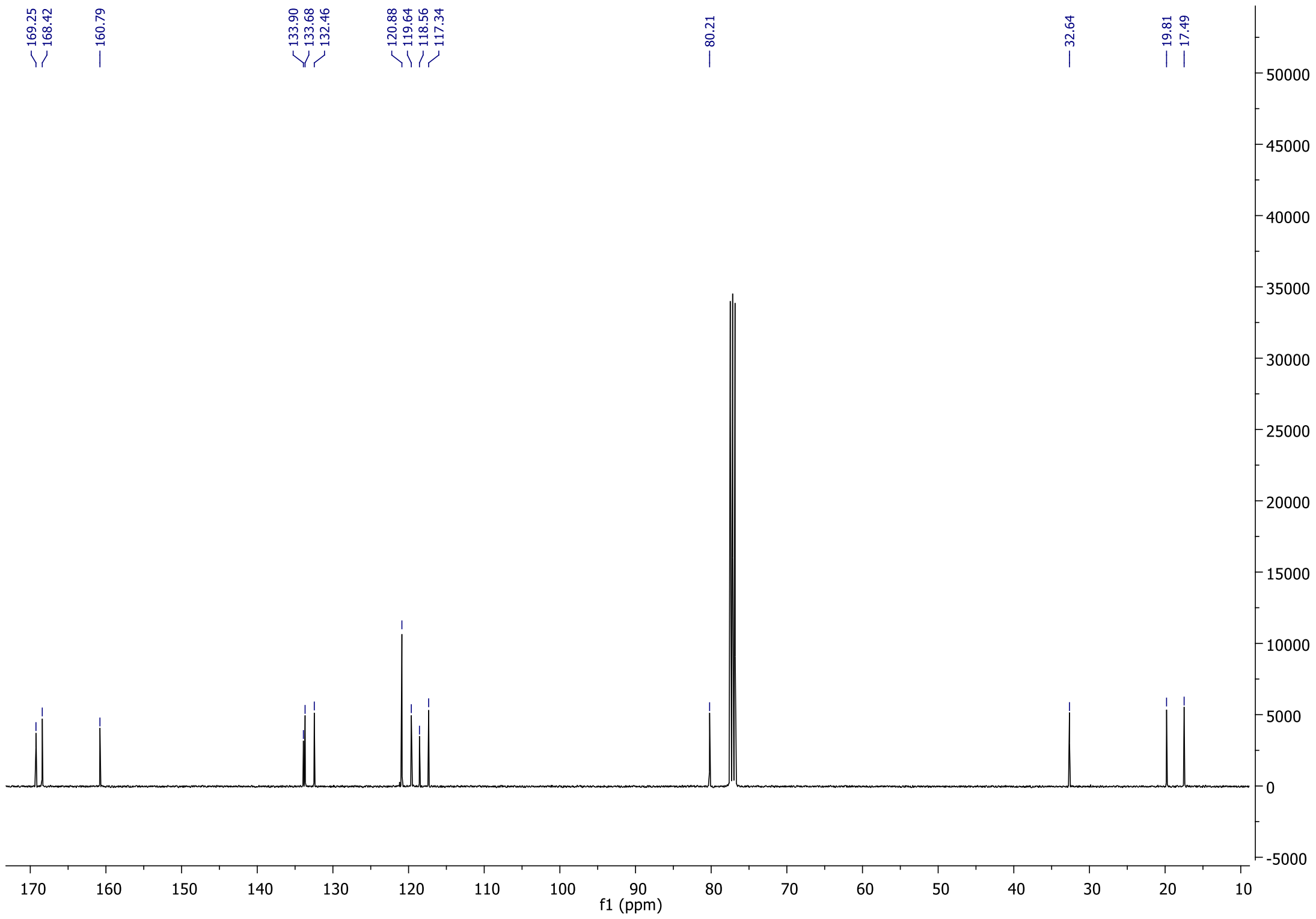

Figure S10. ^{13}C NMR spectrum for **2a** (101 MHz, $CDCl_3$, 5 mM)

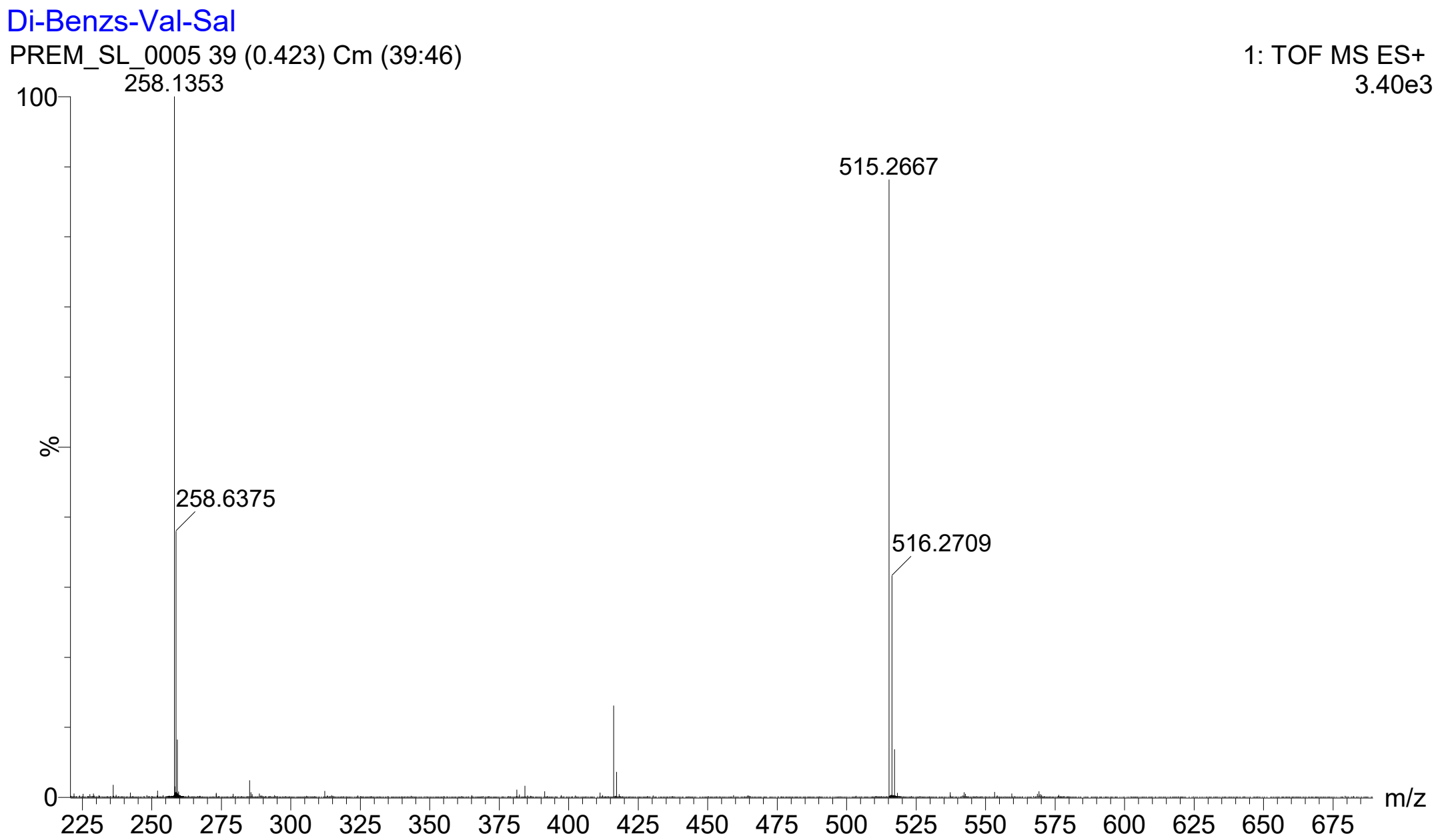

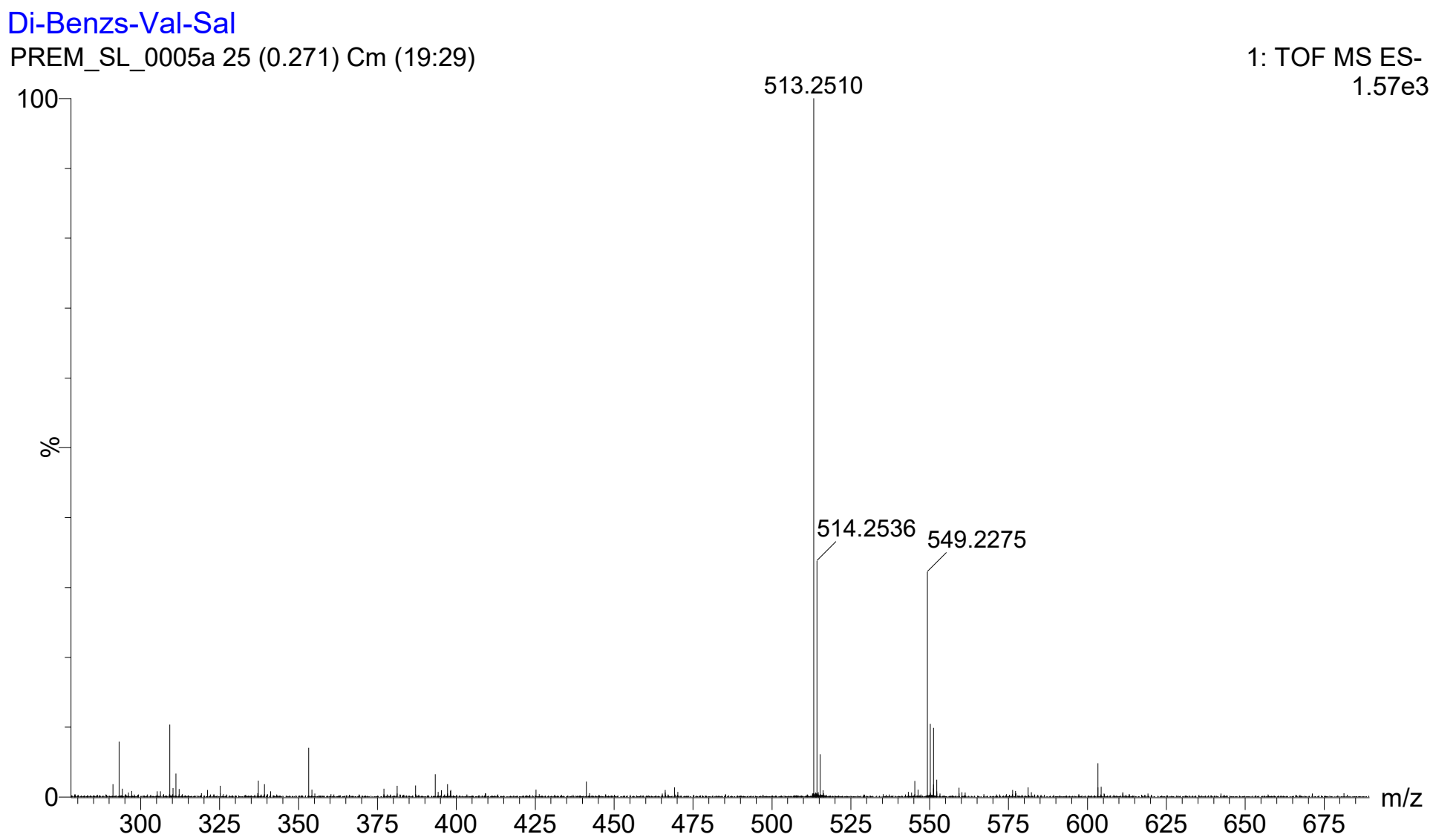

Figure S11. HRMS (ESI-TOF)$^+$ and (ESI-TOF)$^-$ spectra for **2a**

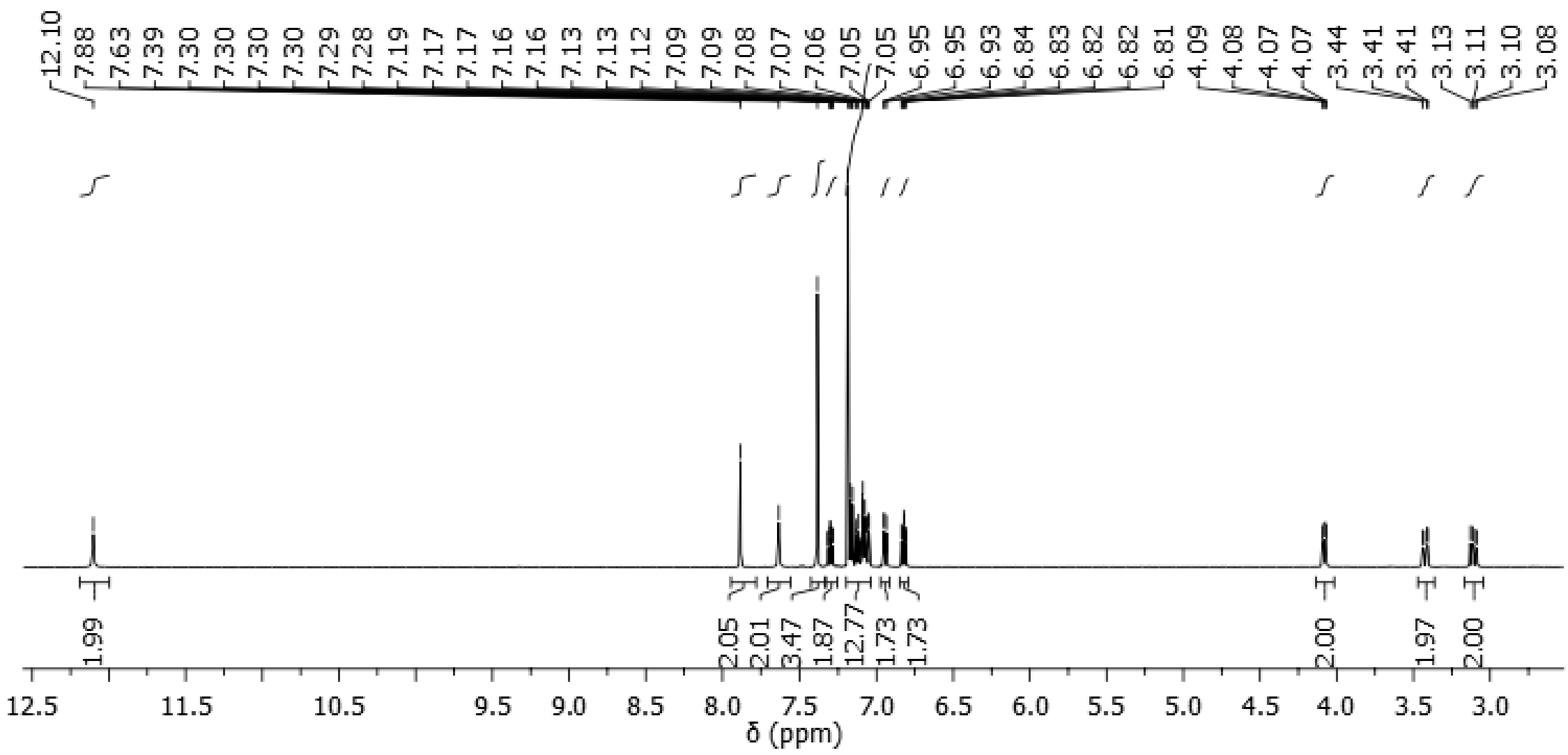

Figure S12. ^{1}H NMR spectrum for **2b** (500 MHz, 0.5 mM, $CDCl_3$))

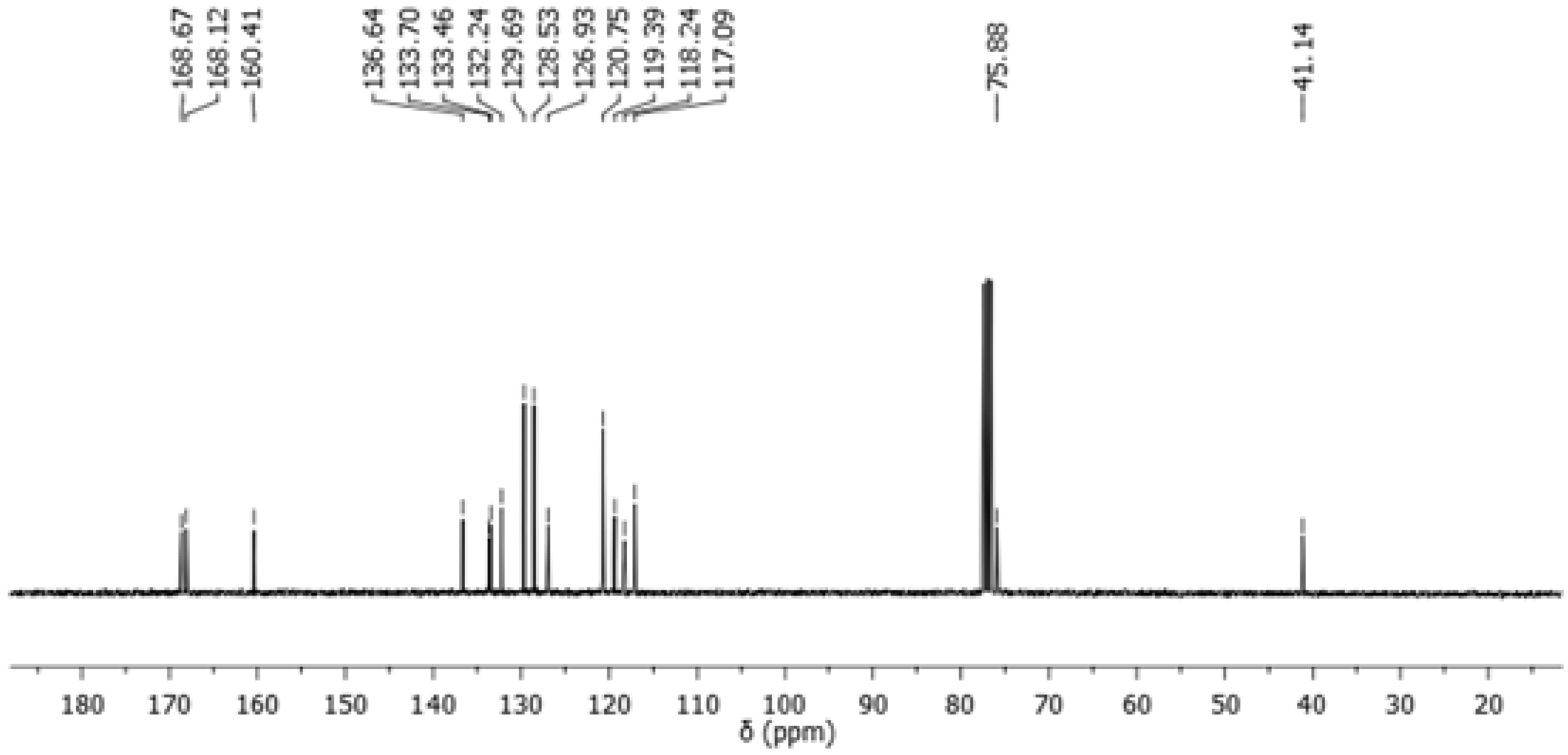

Figure S13. ^{13}C NMR spectrum for **2b** (75 MHz, 0.5 mM, $CDCl_3$)

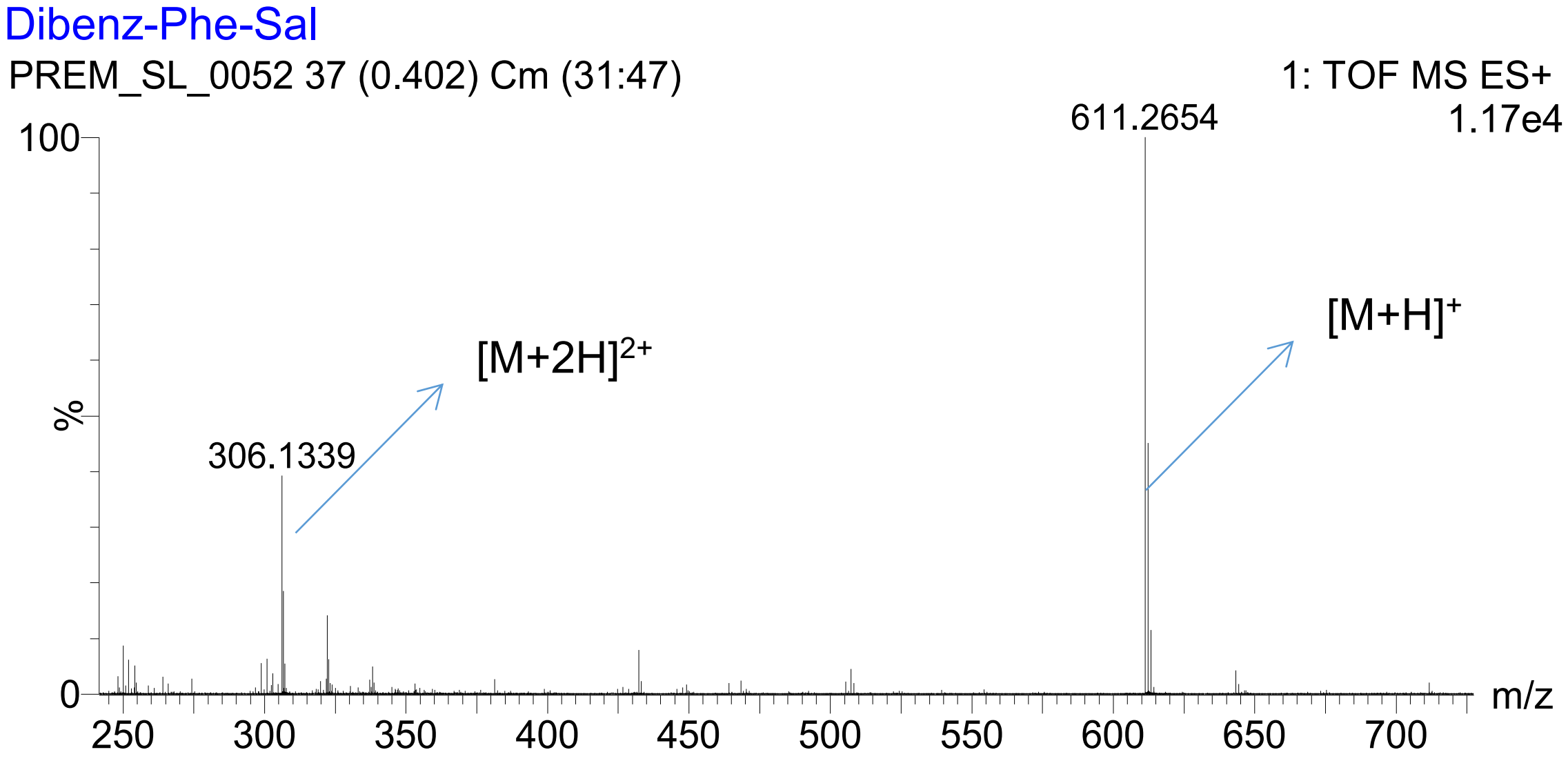

Figure S14. HRMS (ESI-TOF)$^+$spectrum for **2b**

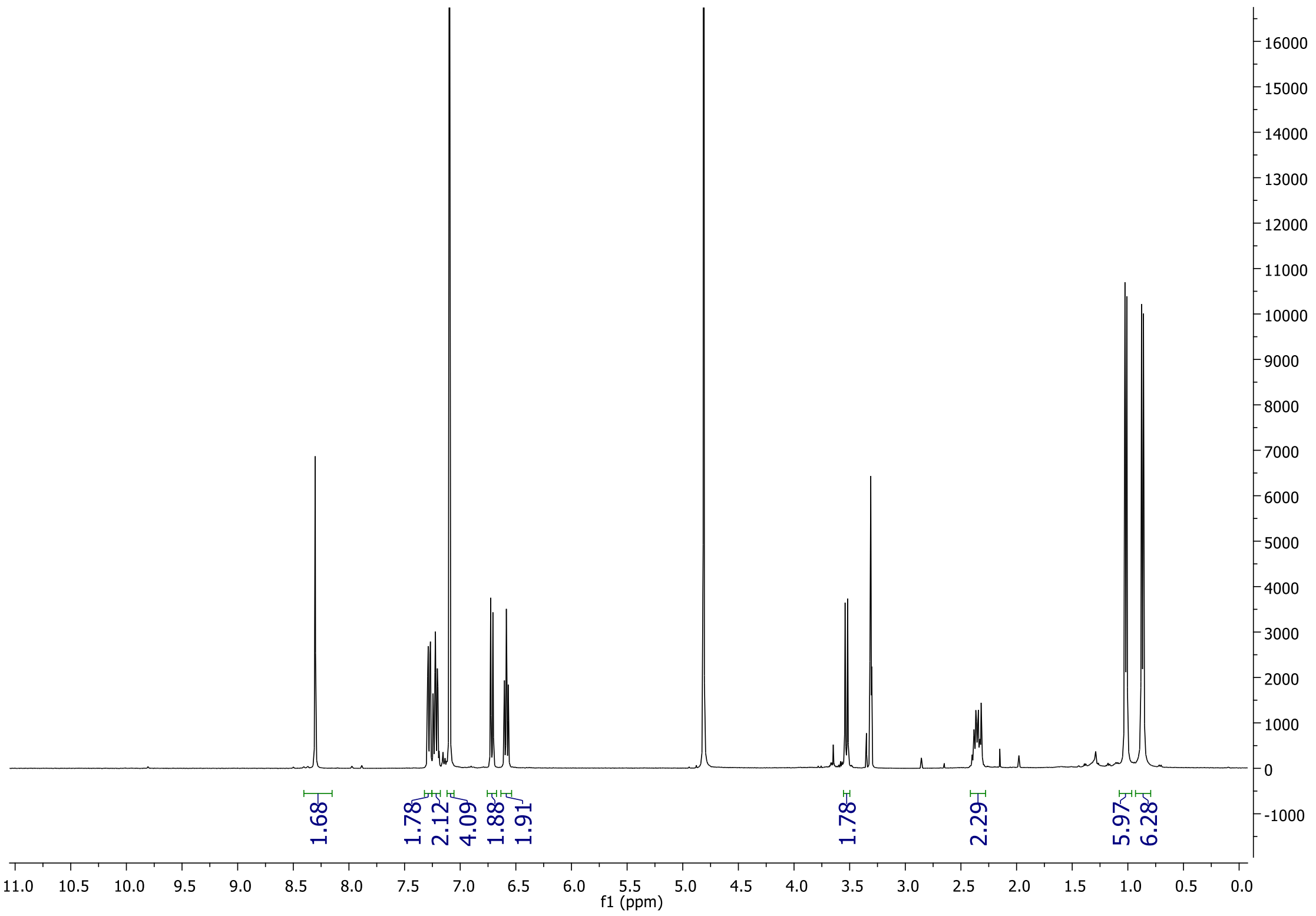

Figure S15. Partial ^1H NMR spectrum for **3a** (400 MHz, CD_3OD, 3 mM):

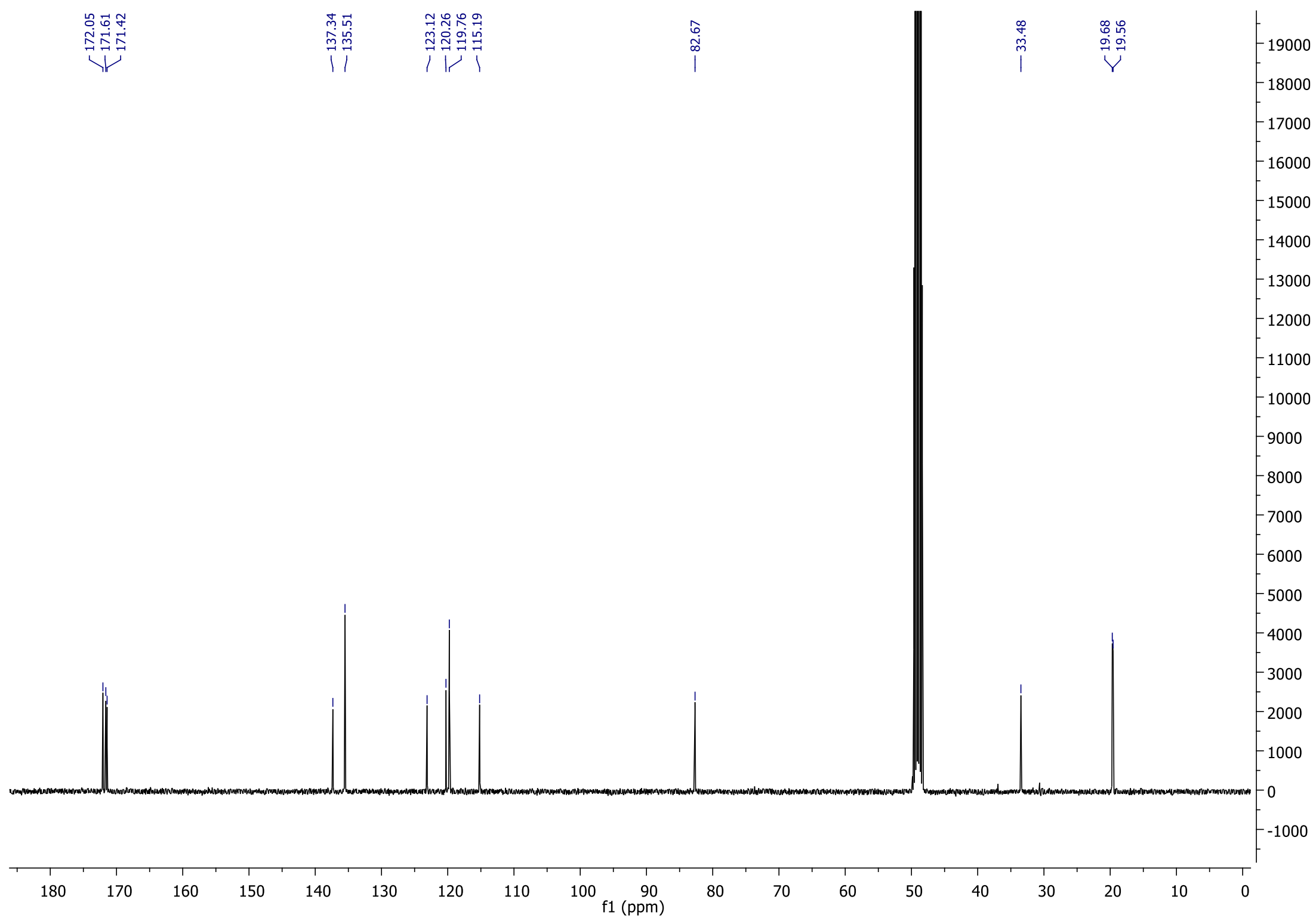

Figure S16. Partial ^{13}C NMR spectrum for **3a** (101 MHz, CD_3OD, 3 mM).

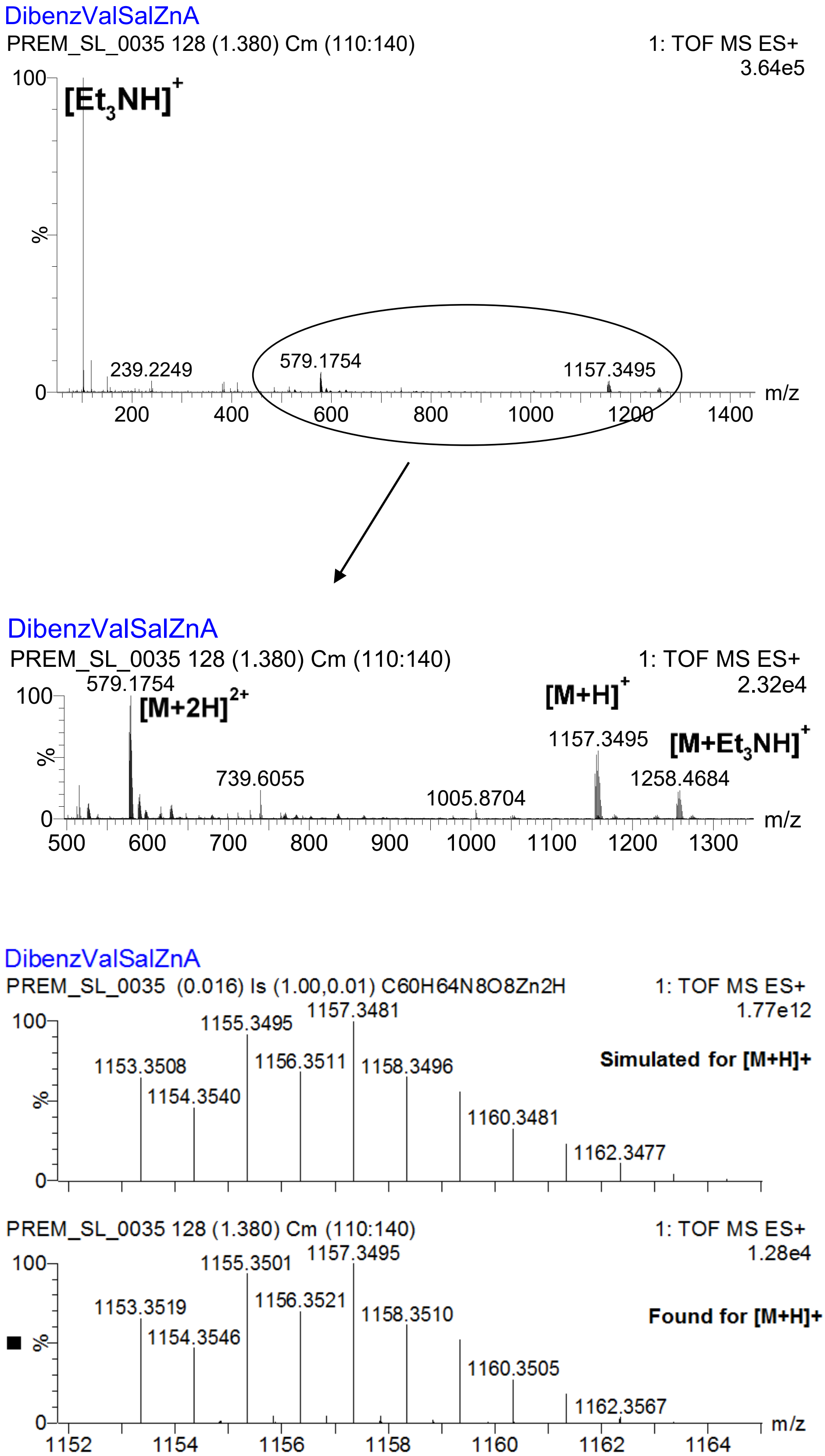

Figure S17. HRMS (ESI-TOF)$^{+}$ spectra of **3a**
